# Holistic Approach for the Comparative Assessment of Chemical Structure and Functional Properties of Major Categories of Agricultural Plastics

**DOI:** 10.3390/polym18131656

**Published:** 2026-07-03

**Authors:** Sarai Agustin Salazar, Paolo Maria Riccobene, Sabrina Carola Carroccio, Fabiana Convertino, Antonis Mistriotis, Christina Pyromali, Andrea Antonino Scamporrino, Evelia Schettini, Giuliano Vox, Pierfrancesco Cerruti

**Affiliations:** 1 Institute of Polymers, Composites and Biomaterials (IPCB), National Research Council (CNR), Via Campi Flegrei 34, 80078 Pozzuoli, Italy; sarai.agustinsalazar@cnr.it (S.A.S.); pierfrancesco.cerruti@cnr.it (P.C.); 2 Institute of Polymers, Composites and Biomaterials (IPCB), National Research Council (CNR), Via Paolo Gaifami 18, 95126 Catania, Italy; paolomaria.riccobene@cnr.it (P.M.R.); andreaantonino.scamporrino@cnr.it (A.A.S.); 3 Department of Soil, Plant and Food Sciences, University of Bari Aldo Moro, Via Amendola 165/A, 70126 Bari, Italy; evelia.schettini@uniba.it (E.S.); giuliano.vox@uniba.it (G.V.); 4 Department of Natural Resources & Agricultural Engineering, Agricultural University of Athens, 75, Iera Odos Str., 11855 Athens, Greece; amistr@aua.gr (A.M.); ch.pyromali@gmail.com (C.P.)

**Keywords:** chemical and functional properties, biodegradable polymers, polyolefins, degradation, mulch, pipe, net

## Abstract

This study evaluates the performance of major types of conventional and bio-based plastic items commonly used in agriculture to provide comprehensive insights into their key structural and functional properties, including the chemical composition of the polymer matrix and additives, mechanical behavior, and thermal and radiometric properties. Twelve agricultural plastic (AP) items were analyzed: covering mulch films, geotextile ground cover, protection fleece and low tunnel fleece cover, fertilizer sack, fly trap, irrigation pipe, tree binding net, guide for tree, silage film and hay bales protection fabric. This selection of APs also encompasses a broad range of basic polymers, including conventional materials (mainly polyethylene and polypropylene) and bio-based formulations (primarily starch- or lignocellulose-containing blends). Mass spectrometry and infrared spectroscopy analyses were performed to assess polymer composition and additives. Mechanical properties were assessed through tensile and puncture tests; in addition, radiometric, thermogravimetric, surface wettability, water absorption and permeability tests were also performed to assess other relevant physical characteristics. The study identified significant differences among bio-based biodegradable APs and compared them with their conventional polyolefin-based counterparts. Material composition and structure were found to critically influence water interactions, shaping the balance between durability, degradation, and crop protection performance. Notably, bio-based mulch films exhibited higher water vapor permeability (0.6–1.1 × 10^−13^ g/m Pa s), reduced penetration resistance (12.1 N) and lowered impact and tensile strengths (21.8 MPa). Water interaction tests showed that the starch-based mulch film displayed very high swelling (above 100%), favoring biodegradation, whereas a biodegradable blend based on polyhydroxybutyrate and polybutylene succinate exhibited minimal swelling (<3%). Material composition and morphology were also key determinants of water vapor transport: dense polymer films provided superior moisture barriers (permeability range 0.013–0.04 × 10^−13^ g/m Pa s), while fibrous or biodegradable materials allowed enhanced vapor permeability. The results of this study, highlighting functionality, advantages and limitations of biodegradable APs versus conventional APs, are intended to guide future innovation in AP design, ensuring alignment with both the operational demands of modern agriculture and environmental sustainability goals. The data obtained from this study can support scientific advancements and policy recommendations on the use and management of plastics in agriculture.

## 1. Introduction

In recent decades, the widespread use of plastics in agriculture has significantly improved this sector, streamlining agricultural activities and providing increased quantity and quality of food production. However, it is imperative to confirm that the considerable short-term benefits provided by plastics do not imply major long-term sustainability problems [1]. To do this, a thorough knowledge of these plastics is a crucial first step.

As is known, conventional plastic is an umbrella term covering countless oil-based materials consisting of polymers and additional compounds, such as stabilizers, antioxidants, ultraviolet (UV) absorbers, pigments, and fillers, that can facilitate the manufacturing process and/or provide the final plastic product with the targeted properties. This applies to all plastic products, and agricultural plastics (APs) are no exception. Among the most widely used APs, mulch films are extensively applied, alongside greenhouse covering films, nets and irrigation pipes, with usage patterns varying according to climatic context and agricultural practices. Inevitably, this results in the generation of a huge amount of agricultural plastic waste (APW) at the end of the useful life of APs [2].

APs based on polyolefins, such as polyethylene (PE) and polypropylene (PP), are considered persistent due to their chemical structure, which makes them non-biodegradable in natural environments. They can fragment into micro- and nano-plastics (MNPs), accumulating in soil and being easily taken up by biota or transported through ecosystems [3]. Therefore, these APs should be recovered after use to limit the accumulation of persistent MNPs in soil and the leaching of the contained chemical additives, which negatively affect soil biota and productivity. Conversely, biodegradable APs are designed and manufactured considering the microbial metabolic process leading to their conversion under aerobic conditions into CO_2_, water and small amounts of microbial mass at end of life.

Biodegradable polymers are generally classified considering origin, chemical composition, synthesis and processing method and application. According to their origin, biodegradable polymers can be natural or synthetic. The former, including polysaccharides (starch, chitin/chitosan, alginate, lignin, etc.) and protein-based polymers (collagen, albumin, gelatin, etc.), are derived from natural sources and have generally high biodegradability [4]. The synthetic polymers, which encompass aliphatic polyesters (e.g., poly(glycolic acid), poly(lactic acid)), polyhydroxyalkanoates, and poly(anhydrides), are more customizable in terms of mechanical performance, heat resistance, and processability, but sometimes at the expense of slower degradability [5]. Concerning production, biodegradable polymers can be obtained by direct processing of natural polymers (chemically and microbiologically synthesized), as well as by enzymatic and chemo-enzymatic synthesis [4].

These materials must pass several tests on chemical composition, complete degradation in laboratory and real-world conditions and ecotoxicity to be qualified as biodegradable [6], as biodegradation depends both on the physico-chemical properties of the product and the environmental conditions of the exposure context [7]. As an example, the biodegradability of products certified as biodegradable in soil, which are intended to be plowed after use [8], is assessed according to recognized international standards. ISO 17556:2019 [9] specifies methods for determining aerobic biodegradation in soil, while UNI EN 13432 [10] defines requirements for biodegradable and compostable materials. UNI EN 17033 [11] establishes specific requirements for mulch biodegradation in soil under field conditions.

The use of APs and the potential release of MNPs into terrestrial ecosystems alter physical, chemical, and biological soil properties. This is attributed to the intrinsic properties of plastic products, their photodegradation process in the field, mainly driven by solar UV radiation, and to their end-of-Life (EoL) management. The chemical composition of the material, morphology, size, and surface characteristics are very relevant factors [3,12], as they influence AP behavior during use and at EoL, as well as the resulting environmental impacts. In this regard, the negative effects associated with the release of plastic particles and related additives into the environment, and subsequently in the food chain, are a critical issue requiring further investigation [13]. The release of MNPs has detrimental consequences for soil properties and microbial communities [14,15], induces physiological and biochemical impacts on flora and fauna [16,17] and contributes to microbiome disruption [18]. Additive leakage represents another major concern. The release timeframe can vary widely, from days (as in the case of more polar phthalates) to centuries (for more hydrophobic additives), and long-term release dynamics in particular require deeper study [19,20]. Moreover, some additives, such as phthalates and bisphenols, are known for their toxic effects [21].

Therefore, the chemical composition of each AP category strongly influences APW management. Reuse and recycling, after proper collection, are preferred [22], but they are only feasible when APs possess suitable physical and chemical characteristics and are free from residues and problematic additives [23]. When APs become too degraded or soiled to be recovered, materials certified as biodegradable in soil should be employed, ensuring complete biodegradability [1,24]. These materials must contain only eco-friendly additives [25] and allow for biodegradation in situ under real environmental conditions, which differ substantially from the controlled laboratory settings defined by standard test methods [26]. To promote a more sustainable approach to APs, more information on AP characteristics and specific chemical composition is needed [27]. Additionally, greater sustainability can be achieved through innovation in material design aimed at producing more environmentally benign products. From this perspective, an inventory and thorough characterization of the most widely used APs can serve as a valuable tool. The overall goal of this paper is therefore to provide in-depth knowledge of representative examples from the main AP categories used in Europe to support informed choices, appropriate use and effective management at their EoL. The APs selected for testing were identified through consultation with multi-stakeholder industry working groups across the agri-plastics value chain and informed by the authors’ pilot surveys. The materials examined include mulch and covering films, as well as other AP types that may contribute to MNP contamination of soils, encompassing both petroleum-based and bio-based plastics in both non-biodegradable and biodegradable forms. To pursue the general objective, the research set specific goals related to the assessment of the chemical, physical, and mechanical properties of selected APs, with the aim of understanding their composition, functionality, and potential environmental impacts. These specific aims were fully achieved through in-depth, targeted analyses. Detailed chemical characterization was performed to assess the polymer matrix composition and to identify possible chemical additives present in the formulations, as AP composition, including both base polymer and additives, is often proprietary information not disclosed by manufacturers. Morphological and functional properties were also investigated, including tensile and puncture behavior, radiometric properties, water absorption, and water vapor permeability. All laboratory analyses were performed on pristine APs; no field trials were conducted, as the overarching goal of the study was the characterization of unused materials.

The output of this work provides comprehensive data on the composition and characteristics of APs. The results are intended to guide future innovation in AP design, ensuring that new materials meet the operational requirements of modern agriculture while aligning with environmental sustainability goals. The data generated in this study can support not only scientific progress but also the development of policy recommendations concerning the use and management of plastics in agriculture.

## 2. Materials and Methods

### 2.1. Collected Samples of Major AP Categories

**Figure 1:**
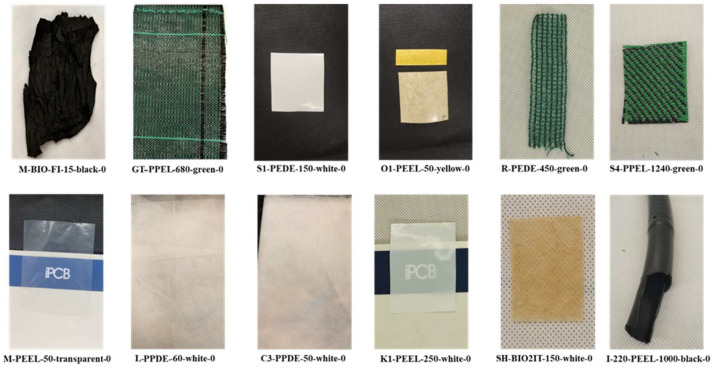
Images of the selected AP samples, representative of the major categories of plastic items used in agriculture.

Pristine commercial samples were collected among 12 of the major AP categories for analysis (Figure 1). These samples of major AP categories were chosen based on their material properties (composition, functional characteristics), market size, and EoL. The 12 AP samples were a heterogeneous group in terms of products, composition and characteristics, but included the most used APs, according to relevant stakeholders. The variety of APs should not be considered a limitation of the research, but rather a strength of this inventory, providing details on all of these products. More detailed information on the samples collected is presented in Table 1. These AP products include 2 mulch films consisting of a conventional linear low-density polyethylene (LLDPE) film and a biodegradable starch/polybutylene adipate terephthalate (PBAT) blend film, 1 low-density polyethylene (LDPE)-based low tunnel fleece, 1 PP-based fleece cover, 1 PP geotextile ground cover, 2 LDPE and PP-based silage/hay bale protection films, 1 LDPE fertilizer sack, 1 LDPE fly trap, 1 LDPE drip pipe, 1 high-density polyethylene (HDPE) tree binding net, and 1 biodegradable polyester-based shelter/guide for vine trees. A taxonomical study of APs was carried out by Briassoulis [28] and Tartiu et al. [29].

### 2.2. Analysis of Polymer Composition and Chemical Additives

#### 2.2.1. Pyrolysis/Thermal Desorption Gas Chromatography–Mass Spectrometry (Py/TD-GC-MS)

To characterize the polymer matrices, Pyrolysis/Thermal Desorption Gas Chromatography–Mass Spectrometry (Py/TD-GC-MS) analyses were performed. A Multi-Shot Pyrolyzer (EGA/PY-3030D, Frontier Labs, Liverpool, UK) was used, coupled to a GC-2020 system (Shimadzu Corporation, Kyoto, Japan) and a triple quadrupole mass spectrometer (TQ8040, Shimadzu Corporation) operating with electron ionization (70 eV). Chromatographic separation was achieved using an Ultra Alloy^®^ metal capillary column (Frontier Labs) with a 5% diphenyl-methylpolysiloxane stationary phase (250 μm inner diameter, 0.25 μm film thickness, 30 m length).

Approximately 0.1 mg of each sample was analyzed. The interface temperatures of the Py-GC and GC-MS systems were set at 300 °C and 250 °C, respectively. To improve spectral interpretation and facilitate the identification of pyrolysis products, a stepwise thermal desorption approach was applied. This method enables the sequential release of volatile and semi-volatile compounds at progressively higher temperatures, enhancing their separation and reducing the resulting chromatogram’s complexity. Specifically, biodegradable materials were analyzed at 300 °C, 400 °C, and 500 °C, whereas non-biodegradable materials were analyzed at 400 °C and 500 °C. This multistep thermal desorption strategy improves peak resolution and helps distinguish the degradation pathways of different polymer fractions.

The GC oven temperature was initially held at 50 °C for 1 min, then increased to 100 °C at a rate of 30 °C/min and maintained for 5 min. It was subsequently ramped to 300 °C at 10 °C/min and held for 10 min. Helium was used as the carrier gas at a constant flow rate of 1.78 mL/min, with a split ratio of 1:50. Blank measurements were performed before each analysis by heating an empty crucible under identical pyrolysis conditions to ensure baseline accuracy. The chemical structures of the pyrolysis products were identified by comparing the obtained mass spectra with reference databases (NIST11.Lib.; NIST11s.Lib.; WILEY8.LIB).

#### 2.2.2. MALDI-TOF Mass Spectrometry

Matrix-Assisted Laser Desorption/Ionization Time-of-Flight Mass Spectrometry (MALDI-TOF MS) was employed to characterize the polymeric matrices present in the selected biodegradable APs, specifically M-BIOFI-15-black-0 and SH-BIO2IT-150-white-0. Spectral acquisition was carried out using an UltrafleXtreme instrument (Bruker Daltonics GmbH & Co, Bremen, Germany) with an accelerating voltage of 20 kV and a delay time of 250 ns. The laser (SmartBeam 2, Bruker Daltonics GmbH & Co, Bremen, Germany) power was set at the minimum threshold required to obtain detectable sample signals, and each spectrum was obtained by collecting 1000 laser shots. Measurements were performed in reflectron mode with positive ion detection. For sample preparation, DHB (0.1 M tetrahydrofuran, THF) was used as the matrix. M-BIOFI-15-black-0 (5 mg/mL in THF) and SH-BIO2IT-150-white-0 (5 mg/mL in hexafluoroisopropanol, HFIP) were prepared, filtered, and concentrated to isolate the soluble fraction. Appropriate volumes of polymer solution and matrix solution were mixed in 2:1, 1:1, and 1:2 ratios (sample/matrix, *v*/*v*). A 1 µL aliquot of each mixture was deposited onto the MALDI-TOF sample plate and air-dried to promote matrix crystallization. To ensure reproducibility, three independent regions of APs were analyzed. Data processing was performed using FlexAnalysis software (Version 3.4). The MALDI-TOF spectra acquired for M-BIOFI-15-black-0 exhibited a resolution (full width at half maximum, FWHM) ranging from 21,000 to 26,000, with mass accuracy between 100 and 150 ppm within the *m*/*z* range of 800–2000 Da. For SH-BIO2IT-150-white-0, the FWHM ranged from 13,000 to 24,000, and mass accuracy varied between 160 and 200 ppm within the same *m*/*z* range (800–2000 Da).

#### 2.2.3. Inductively Coupled Plasma Mass Spectrometry (ICP-MS)

To determine the total concentrations of metals in the polymer samples, an acid digestion procedure was employed, followed by analysis using inductively coupled plasma mass spectrometry (ICP-MS). Approximately 200 ± 1 mg of each sample was subjected to microwave-assisted digestion using an Anton-Paar Multiwave 3000 system (Anton-Paar, Graz, Austria), with digestion conducted in PTFE vessels according to EPA Method 3052 [31]. Each digestion involved the addition of 3.5 mL of concentrated HNO_3_ and 1.5 mL of H_2_O_2_ to facilitate breakdown of the sample matrix. After digestion, the solutions were diluted with 5 mL of deionized water and filtered through Whatman 40 filters to remove particulates. A 1000 µL aliquot of the resulting filtrate was then diluted to 50 mL to prepare the samples for metal quantification using a PerkinElmer Nexion 300X ICP-MS instrument (PerkinElmer, Bridgeport Ave Shelton, CT, USA), following EPA Method 6020 [32]. Results were normalized with mineralization and measurement blanks and are reported in mg/kg. Each determination was performed in triplicate, with a relative standard deviation (RSD) below 10%.

#### 2.2.4. Attenuated Total Reflection Fourier Transform Infrared Spectroscopy (ATR-FTIR)

FTIR analysis was acquired in Attenuated Total Reflection mode (ATR-FTIR) on the AP samples using a PerkinElmer Spectrum 100 (Waltham, MA, USA) equipped with a diamond/ZnSe crystal (1 Reflection) UATR accessory. Transmittance spectra were recorded as the average of 16 scans (range: 4000–650 cm^−1^, resolution: 4 cm^−1^). The ATR crystal was fully covered by the sample, and the pressure device available on the instrument was used to ensure contact between the sample and the ATR crystal.

### 2.3. Mechanical Characterization

#### 2.3.1. Tensile Tests

The tensile properties of the AP materials studied were evaluated using an Instron 5900 Series dynamometer (Instron, Norwood, MA, USA) under controlled conditions of 23 ± 1 °C and 50% relative humidity (RH). The procedures followed were in strict accordance with the ISO 527-1:2018 [33] standard for determining the tensile properties of thin plastic films properly adapted to nets. This standardized approach ensured the accuracy and comparability of the tensile strength, elongation, and modulus data obtained for each AP type.

#### 2.3.2. Puncture Tests

Puncture testing primarily involves the evaluation of tear and stiffness behavior and measures the resistance of a test specimen to puncture. Puncture resistance is a key property of flexible films and membranes; for example, in agricultural applications, mulching films are often exposed to damage caused by the penetration of weeds.

To assess the resistance of APs to the localized pressure exerted by the hard tips of spontaneous weeds, puncture tests were performed on film- and membrane-shaped samples, such as M-PEEL-50-transparent-0, M-BIOFI-15-black-0, SH-BIO2IT-150-white-0, S4-PPEL-1240-green-0, K1-PEEL-250-white-0, and O1-PEEL-50-yellow-0. Circular samples with a 40 mm diameter were clamped using a metal ring on top of an aluminum cup, which was gripped to the lower specimen holder of an INSTRON 4023 dynamometer (Instron, Norwood, MA, USA). The material was then compressed by a spherical steel probe mounted on the moving crosshead, which was then driven downward at a constant rate of 2 mm/min until material rupture occurred. The applied load as a function of displacement was recorded. All mechanical tests were performed on at least three specimens, previously conditioned at room temperature and 50% air RH.

### 2.4. Radiometric Test

Laboratory tests to evaluate the radiometric properties of the materials were carried out using spectrophotometers. A double-beam ultraviolet–visible–near-infrared (UV-VIS-NIR) spectrophotometer (Lambda 950, PerkinElmer Instruments, Norwalk, CT, USA) was used to measure the spectral direct transmissivity of the materials across the solar wavelength range (200–2500 nm). Measurements were performed in 10 nm steps using radiation with a direct perpendicular incidence. Spectral total transmissivity and reflectivity were measured using an integrating sphere (diameter 100 mm) that served as the receiver of the Lambda 950 spectrophotometer, using a double-beam comparative method.

A Fourier Transform Infrared (FT-IR) spectrophotometer (1760 X, PerkinElmer Instruments, Norwalk, CT, USA) was used to measure spectral transmissivity and reflectivity in the long-wave infrared radiation (LWIR) range (2500–25,000 nm) in steps of 4 cm^−1^. Spectral transmissivity was measured using radiation with direct perpendicular incidence, while reflectivity was measured at near-normal incidence (10°). Spectral emissivity was calculated from the spectral transmissivity and reflectivity by means of Kirchhoff’s law.

Transmissivity and reflectivity tests in both the solar and LWIR ranges were performed on five samples (40 mm × 40 mm) for each material.

The radiometric coefficients of the materials were calculated for different wavelength bands: the solar wavelength range (200–2500 nm), the photosynthetic active radiation range (PAR, 400–700 nm), and the LWIR range (7500–12,500 nm). The radiometric coefficients in the solar wavelength band were calculated as the weighted average value of the spectral transmissivity using the spectral distribution of the solar radiation at ground level as the weighting function. The transmissivity and emissivity coefficients in the LWIR range were calculated as average values of the spectral transmissivity and emissivity in the wavelength range 7500–12,500 nm.

### 2.5. Thermogravimetric Analysis

Thermogravimetry (TG) measurements were performed using a PerkinElmer Pyris 1 thermo-balance (Perkin Elmer, Milan, Italy) to analyze the weight loss of samples. Approximately 4.5 ± 0.5 mg of each sample was placed in open ceramic crucibles and heated from 25 to 650 °C at a 10 °C/min rate. The tests were performed under an inert atmosphere, with a nitrogen flow rate of 30 mL/min.

### 2.6. Surface Wettability: Water Contact Angle Measurements

Water Contact Angle (WCA) measurements were performed at room temperature using the sessile drop technique and a contact angle system. The First Ten Angstroms FTA1000 Instrument (Portsmouth, VA, USA) was equipped with a high-resolution video camera (752 × 582 pixels and image acquisition speed of 25 frames per second); the data were analyzed by RTA32 software (v2.0, provided by FTA). Measurements followed the static method at room temperature (θ, deg). Briefly, three drops of distilled water (2 μL each) were deposited onto the surface specimens using a micropipette. Afterward, the contact angle was immediately calculated by measuring the angle between the base line of the drop and the tangent.

### 2.7. Water Absorption and Swelling Ratio

Before testing, the samples were subjected to different washing cycles to remove soil residues firmly attached to their surfaces. To calculate swelling, square strips of AP samples (1 cm × 1 cm) were weighed at time zero (m_1_) and immersed in 20 mL of deionized water for different durations (0, 1, 2, 3, and 5 h). The residual water on the sample surface was carefully wiped off, and the sample was then weighed (m_2_). The swelling ratio was calculated using the following equation:

(1) $$Swelling\;ratio\;(\%)\;=\;(m_{2}-m_{1})/m_{1}\times 100$$

### 2.8. Permeability Tests

Water vapor transmission rate (WVTR) was measured according to the ASTM E96 (1993) [34] wet method, using CEAST (Norwood, MA, USA) aluminum diffusion cell cups with a 6.154 cm^2^ exposed area. The cups were filled with distilled water (RH = 100%) and placed in an environmental chamber set at 25 °C and 50% RH. WVTR values, expressed in g h^−1^·m^−2^, were determined from a linear plot of weight change vs. time, following the equation:

(2) $$WVTR=\frac{∆G}{\left(t*A\right)}$$

where ∆G (g) is the weight change, t (h) is the time during which ∆G occurs, A (m^2^) is the test area of the cell cup, and ∆G/t is the slope of the linear plot.

Water vapor permeability (WVP, in g m^−2^ Pa^−1^ s^−1^) was calculated according to the following equation:

(3) $$WVP=\frac{\left(WVTR*Th\right)}{∆P}$$

where Th (m) is the thickness of the films and ∆P (Pa) is the water pressure difference between both sides of the film. Data were the mean of three measurements for each sample.

### 2.9. Statistical Analysis

Experimental data were expressed as mean ± standard deviation (SD). One-way analysis of variance (ANOVA) was used to evaluate differences among the studied parameters. Significant differences between means were identified using Tukey’s post hoc multiple comparison test at a significance level of *p* < 0.05. All statistical analyses were performed using OriginPro 8.5 (OriginLab Corporation, Northampton, MA, USA).

## 3. Results and Discussion

### 3.1. Composition of the Tested APs

#### 3.1.1. Pyrolysis/Thermal Desorption Gas Chromatography–Mass Spectrometry (Py/TD-GC-MS) and MALDI-TOF

The chemical composition of the 12 selected AP materials was first analyzed using a combination of advanced analytical techniques, including chromatographic, spectrometric and spectroscopic techniques. Pyrolysis/Thermal Desorption Gas Chromatography–Mass Spectrometry (Py/TD-GC-MS) was employed to determine the thermal degradation products characteristic of each AP, reflecting both the macromolecular nature and the volatile additives. Table 2 summarizes the key findings from the Py/TD-GC-MS analyses conducted at 300 °C, 400 °C, and 500 °C. The decomposition products identified at 400 °C and 500 °C provided insight into the composition of the polymer matrix, whereas additive identification was primarily achieved at 300 °C. Compound assignment was performed by comparing the experimental mass spectra with those in the NIST11s and Wiley8 libraries, considering only plausible degradation products with a match score above 85%.

**Figure 2:**
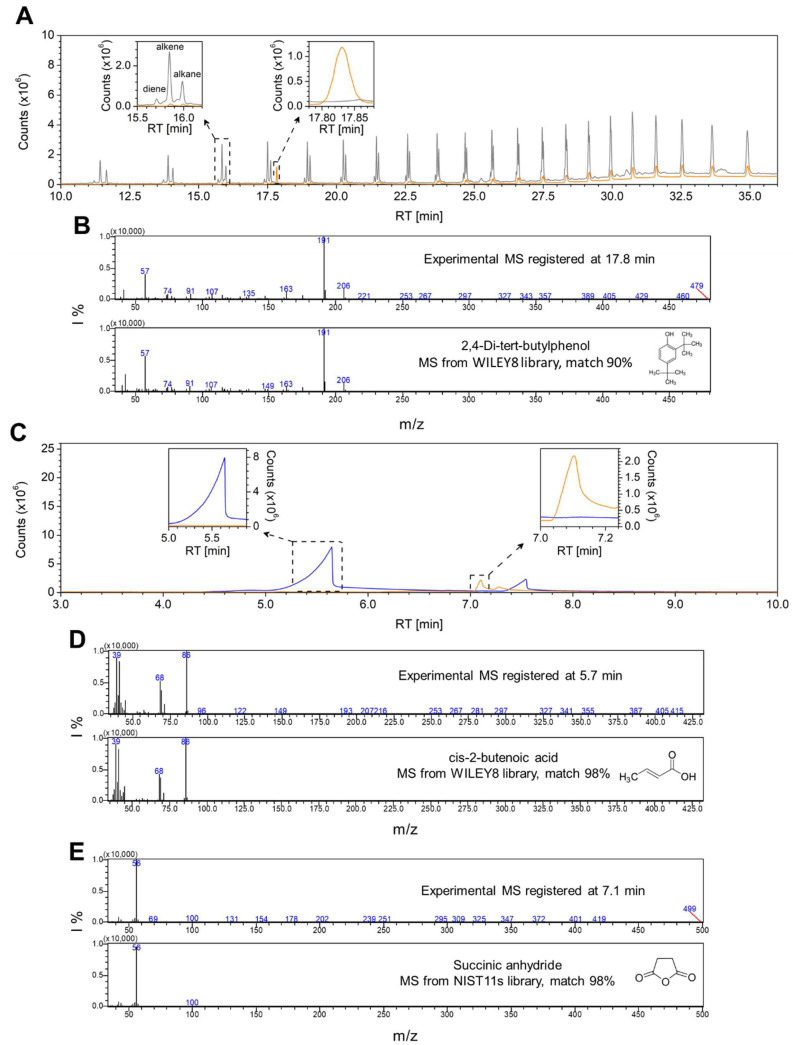
Py/TD-GC-MS results obtained for R-PEDE-450-green-0 and SH-BIO2IT-150-white-0. (**A**) Chromatogram of R-PEDE-450-green-0 at 400 °C (orange line) and 500 °C (gray line). (**B**) Experimental mass spectrum of 2,4-Dimethyl-1-heptene compared with the WILEY8 MS library. (**C**) Chromatogram of SH-BIO2IT-150-white-0 at 300 °C (blue line) and 400 °C (orange line). (**D**) Experimental mass spectrum of cis-2-butenoic acid compared with the WILEY8 MS library. (**E**) Experimental mass spectrum of succinic anhydride compared with the WILEY8 MS library.

Significant differences were observed between conventional polymers, such as R-PEDE-450-green-0, and biodegradable polymers, such as SH-BIO2IT-150-white-0 (Figure 2). The chromatographic profiles of R-PEDE-450-green-0 (Figure 2A) at 400 °C (orange line) and 500 °C (gray line) show distinct degradation patterns characteristic of PE. Notably, the pyrolysis profile features the well-known repeating sequence of paraffin triplets (diene–alkene–alkane) per carbon number, as reported in the inset between RT 15.5 min and 16.5 min. Additionally, at RT 17.7 min, a peak corresponding to 2,4-di-tert-butylphenol, a commonly used antioxidant and UV absorber, is detected. The presence of this additive was confirmed through mass spectral comparison with the Wiley8 MS library, yielding a 90% match (Figure 2B). The chromatograms of SH-BIO2IT-150-white-0 (Figure 2C) reveal the presence of cis-2-butenoic acid (RT 5.5 min) and succinic anhydride (RT 7.1 min), both identified with 98% spectral matching to libraries (Figure 2D,E). This suggests that this AP is composed of biodegradable polyesters. Cis-2-butenoic acid is a well-documented pyrolysis product of polyhydroxyalkanoates (PHAs), while succinic anhydride is a characteristic degradation product of polybutylene succinate (PBS). The simultaneous detection of these compounds suggests that SH-BIO2IT-150-white-0 contains a blend of PHAs and PBS, consistent with formulations designed to balance mechanical performance with environmental degradability.

**Figure 3:**
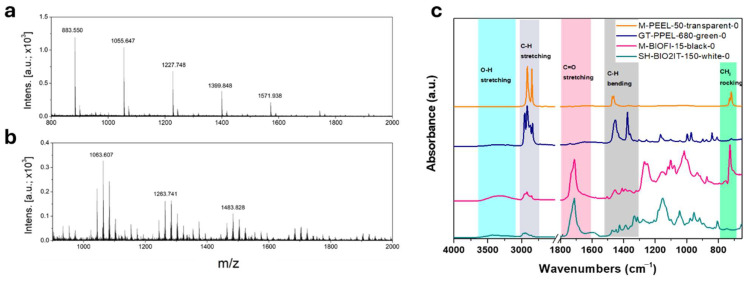
MALDI-TOF spectrum of (**a**) M-BIOFI-15-black-0, and (**b**) SH-BIO2IT-150-white-0 polymer fractions, recorded in positive reflectron mode. (**c**) FTIR-ATR spectra of: M-PEEL-50-transparent-0, GT-PPEL-680-green-0, M-BIOFI-15-black-0, and SH-BIO2IT-150-white-0.

MALDI-TOF analysis provides additional confirmation of the material’s composition, complementing the findings obtained through PY/TD-GC-MS regarding the polymeric nature of the biodegradable plastics. It is important to note that MALDI-TOF preferentially ionizes and desorbs low masses in polydisperse polymeric systems [35]. For the M-BIOFI-15-black-0 material, the resulting spectrum (Figure 3a) confirmed the presence of poly(butylene adipate-co-terephthalate) (PBAT) as the main polymeric constituent.

In particular, the detected mass peaks correspond to sodium adducts of cyclic copolymeric species, whose co-monomer ratio (A/T) and mass-to-charge (*m*/*z*) values are detailed in Table S1. In contrast, the analysis conducted on SH-BIO2IT-150-white-0 (Figure 3b) revealed the presence of poly(butylene succinate) (PBS) as a polymeric constituent, as shown in Table S2. The polyalkenoates identified through Py-GC-MS analysis were not observed in the MALDI-TOF spectrum. This lack of information in MALDI-TOF data is a common occurrence, as the MALDI-TOF process often suffers from polymer preferential desorption depending on the blend formulation.

#### 3.1.2. Inductively Coupled Plasma Mass Spectrometry (ICP-MS)

Inductively Coupled Plasma Mass Spectrometry (ICP-MS) was employed to complement the compositional characterization by quantifying metal ion concentrations in the samples, providing additional insights into their composition and potential environmental impact (Table 3). Each sample was analyzed for the total content of 15 metals: antimony (Sb), mercury (Hg), lead (Pb), aluminum (Al), chromium (Cr), cobalt (Co), copper (Cu), zinc (Zn), arsenic (As), tin (Sn), vanadium (V), cadmium (Cd), iron (Fe), manganese (Mn), and nickel (Ni). These elements were selected based on their possible occurrence in plastics, either as intentionally incorporated (as additives or catalysts during polymer synthesis) or as unintentional contaminants introduced during manufacturing, processing, use, and management [36]. Several of these metals—such as As, Cd, Co, Cr, Hg, and Pb—are of particular toxicological concern due to their classification as carcinogenic, mutagenic, reproductive toxicants, or environmentally hazardous substances [36]. The results listed in Table 3 indicate that metal concentrations in the analyzed samples were generally low, with all values either below detection limits for hazardous elements or within permissible regulatory thresholds. However, a higher concentration of Al was consistently detected in colored samples compared with colorless ones, likely due to the presence of carbon black or pigments, which contribute to the material’s colorimetric properties. A similar trend was observed for Fe. Zn was also detected, potentially originating from phenyl-phosphonic acid zinc salt, a commonly used additive in plastic manufacturing that enhances crystallization and mechanical properties in various polymers [26].

#### 3.1.3. Fourier Infrared (FTIR) Spectroscopy

FTIR spectroscopy enabled the identification of the main functional groups present in the polymers constituting the APs and provided information on some of the additives used (Figure S1). The main absorption peaks observed in the analyzed APs are reported in Table 4, while Figure 3c shows the FTIR-ATR spectra of four representative samples, which are discussed in further detail. In the two polyolefin-based materials (the mulching film M-PEEL-50-transparent-0 and the ground cover GT-PPEL-680-green-0), strong absorption peaks at 2915 and 2848 cm^−1^ are noticed, due to asymmetric and symmetric C–H stretching vibrations, respectively. Additional peaks at 1466 and 1365 cm^−1^ are associated with the combined bands of CH_2_ and CH_3_ scissoring vibration. The spectra of the PE-based APs also show the absorption peak at 725 cm^−1^, due to C–H rocking vibrations in repeating –CH_2_– units [37]. Both materials exhibit a weak and broad absorption band between 1700 and 1550 cm^−1^, attributed to C=C stretching vibrations, likely arising from phenol-based stabilizing additives; this interpretation is supported by the absorption band around 3350 cm^−1^, typical of hydroxyl group stretching [38].

For the biodegradable AP samples, the mulching film M-BIOFI-15-black-0 shows the presence of bands attributable to PBAT and starch. The peak around 1020 cm^−1^ is due to the C–O stretching vibrations associated with the glycosidic part of the starch, more specifically, the glycosidic bonds between glucose units. Further, the characteristic PBAT functional groups are visible at 3350 cm^−1^ (OH stretching), 3000 cm^−1^ (C–H stretching of aliphatic and aromatic fractions), 1715 cm^−1^ (C=O carbonyl groups) and 1270 cm^−1^ (C–O in ester linkages). Additional bands at 1578, 1456, 1409 and 1017 cm^−1^ are assigned to the stretching of phenylene groups, whereas the sharp band located at 726 cm^−1^ is characteristic of four or more adjacent methylene groups. Furthermore, bending modes of substituted benzene are recognized in the 700–900 cm^−1^ spectral range [39]. The FTIR-ATR spectrum of the tree shelter SH-BIO2IT-150-white-0 also displays absorption peaks characteristic of a polyester matrix, including OH stretching around 3350 cm^−1^, carbonyl stretching at 1715 cm^−1^, and the stretching vibration of C–O at 1045 cm^−1^ [40]. However, the lack of features in the region around 730 cm^−1^ suggests the absence of adjacent –CH_2_– groups in the main chain, corroborating the presence of a polyhydroxyalkanoate (PHA) or a polysuccinate (PES, PBS) as a polymer matrix, as suggested by Py-GC-MS analysis. A prominent peak occurring in the 1120–1160 cm^−1^ wavenumber region, typical of polysaccharide C–O–C stretching, indicates the introduction of lignocellulosic biomass as a filler in the polymer formulation [41].

### 3.2. Functionalities of the Tested APs

#### 3.2.1. Mechanical Properties

##### Tensile Properties

The tensile test results for the selected APs (Table 5) reveal clear differences in mechanical performance across product categories. Conventional PE-based films, such as M-PEEL-50-transparent-0, exhibit the highest tensile strength and elongation values among the mulching films, reflecting their high ductility and ability to undergo substantial plastic deformation before rupture. In contrast, the biodegradable film M-BIOFI-15-black-0 shows lower tensile strength and reduced elongation, indicative of a more brittle behavior typical of biodegradable mulching films of comparable thickness. The tube I-220-PEEL-1000-black-0 displays both high tensile strength and considerable elongation at break, confirming robustness and ductility required for irrigation applications. Other conventional films, such as those used for fertilizer sacks and silage wrapping (K1-PEEL-250-white-0 and S1-PEDE-150-white-0), also exhibit relatively high tensile strength and elongation, supporting their suitability for mechanical handling and load-carrying capacity. In contrast, the fly trap film (O1-PEEL-50-yellow-0) presents lower elongation and moderate strength, consistent with its intended function. Nonwoven and geotextile-type products, such as GT-PPEL-680-green-0 and C3-PPDE-50-white-0, show significantly lower elongation but comparatively higher tensile strength per unit width. This behavior reflects their design priorities, i.e., dimensional stability and tear resistance rather than ductility. Similarly, bale nets and protective fabrics (e.g., S4-PPEL-1240-green-0) combine high tensile strength with moderate elongation, providing a balance between mechanical integrity and controlled elasticity. Finally, biodegradable materials intended for temporary applications, such as SH-BIO2IT-150-white-0, display much lower tensile strength and minimal elongation, confirming their brittleness compared to their conventional counterparts. Overall, the results demonstrated that PE- and PP-based materials maintain superior mechanical strength and ductility, whereas biodegradable alternatives are more suitable for short-term applications where the EoL biodegradability is the primary requirement.

##### Puncture Resistance

The puncture behavior of the selected mulching films, ground cover films, and young tree shelter was evaluated by recording the maximum load (N) as a function of displacement (mm) (Table 6). In general, puncture properties of materials are proportional to the thickness of the specimens and depend on their polymeric composition. For a given material, thicker samples exhibit higher elongation at puncture, as observed for K1-PEEL-250-white-0 (Table 6) [42]. This behavior is particularly relevant for fertilizer sacks, which must withstand handling and transportation stresses; high puncture resistance combined with high displacement capacity helps prevent sudden tearing under load. Similar trends are observed for PE films such as O1-PEEL-50-yellow-0 and M-PEEL-50-transparent-0 (made of LLDPE), which show comparatively higher load values. This enhanced resistance contributes to durability in applications where these products must tolerate environmental exposure without easily tearing. Conversely, the PP-based hay bale protection film S4-PPEL-1240-green-0 exhibits good ductility but lower puncture strength, indicating a highly flexible material with limited resistance to sharp objects. M-BIOFI-15-black-0 has significantly lower puncture resistance (12.1 N) and shorter displacement (14.7 mm). The lower thickness (15 µm) and biodegradable nature likely contribute to its reduced mechanical performance compared to PE-based mulch films. This behavior reflects the intrinsic brittleness or the different cohesive forces of the biodegradable blend, made up of PBAT and thermoplastic starch (TPS), compared to the more homogeneous PE matrix.

Finally, SH-BIO2IT-150-white-0 exhibits very low puncture resistance (2.4 N) and short displacement (12.9 mm). Despite its thicker structure (150 µm), this biodegradable blend of polymers has very limited elongation at break and pronounced brittleness, making it more prone to puncture damage. Its minimal displacement at failure further reflects its low capacity for plastic deformation. However, such behavior may be acceptable if the material is designed to provide temporary protection to young trees before gradually degrading in the environment.

#### 3.2.2. Radiometric Properties

The radiometric coefficients of the materials in the solar, PAR, and LWIR ranges are shown in Table 7. The main radiometric requirement of a black mulching film is to be opaque to PAR radiation; a very low PAR transmissivity prevents the passage of solar radiation in wavelengths essential for photosynthesis, thereby reducing weed development [43]. The tested black mulching film (M-BIOFI-15-black-0) showed a PAR transmissivity coefficient of 1.7%.

Transparent mulch films are designed to increase soil temperature by allowing solar radiation to pass through the material. The higher the solar transmissivity coefficient, the greater the soil warming effect under the mulch. The transparent mulching film (M-PEEL-50-transparent-0) showed a high solar total transmissivity coefficient, equal to 89.6%. The LWIR transmissivity coefficient also plays a crucial role in soil temperature regulation. The lower the LWIR transmissivity coefficient of the mulching film, the higher the soil temperature under the mulching film. Among the tested mulching films, M-BIOFI-15-black-0 was the most effective in limiting radiative heat loss, with the lowest LWIR transmissivity coefficient (equal to 16.2%). The other materials displayed more variable radiometric behavior, reflecting differences in polymer composition, thickness, pigmentation, and intended application.

One of the primary functions of a geotextile used as ground cover is to prevent the growth of spontaneous plants; thus, a very low PAR total transmissivity coefficient is desirable. The ground cover film (GT-PPEL-680-green-0) meets this requirement, exhibiting a very low PAR total transmissivity coefficient, equal to 0.2%, indicating minimal penetration of PAR.

The fertilizer sack K1-PEEL-250-white-0 showed a PAR total transmissivity coefficient equal to 45.3%, whereas the silage sack S1-PEDE-150-white-0 showed a lower value, equal to 27.6%. Silage bags provide a cost-effective and secure method for the temporary storage of maize, grass, grain, and other crops. They create airtight conditions that promote optimal fermentation and preserve nutrients, even when the stored material has a moderate moisture content. Their opaque barrier helps limit solar radiation exposure, thereby reducing photodegradation and inhibiting unwanted biological activity. This explains the lower PAR transmissivity of silage sacks, which contributes to maintaining feed quality and preventing spoilage.

The protection fleece (C3-PPDE-50-white-0), which is not a black material, is characterized by high solar and PAR total transmissivity coefficients, equal to 87.1% and 86.8%, respectively. Such values are consistent with its intended function.

#### 3.2.3. Morphological Characterization

**Figure 4:**
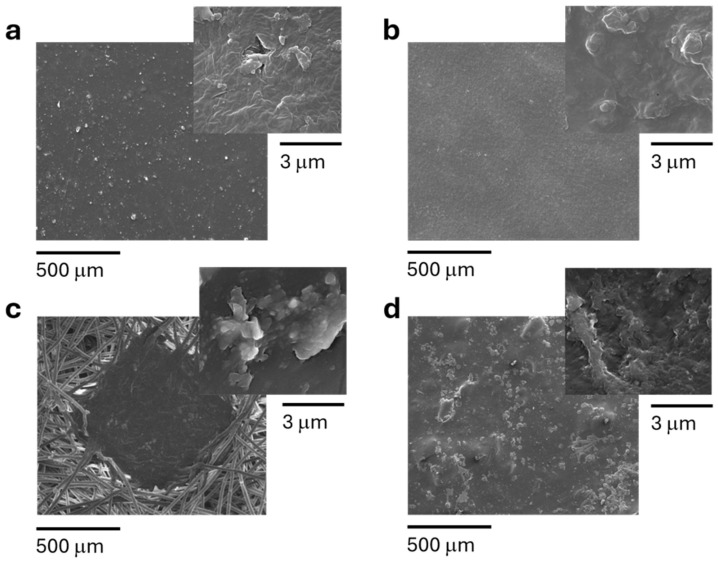
SEM images of the surface of: (**a**) M-PEEL-50-black-0, (**b**) M-BIOFI-15-black-0, (**c**) C3-PPDE-50-tranparent-0, and (**d**) SH-BIO2IT-150-white-0 at 200× and 40,000× (inset) magnification.

The morphological characterization of the two mulch films (M-PEEL-50-transparent-0 and M-BIOFI-15-black-0), the protection fleece (C3-PPDE-50-white-0), and the shelter (SH-BIO2IT-150-white-0) was carried out by SEM microscopy, and the corresponding surface micrographs are shown in Figure 4. At low magnification (200×), M-PEEL-50-transparent-0 shows a smooth texture, indicative of quite homogeneous materials. However, small particles are visible on the film surface, attributable to the presence of fillers (Figure 4a). Higher magnification images (Figure 4a, inset) also highlight the presence of surface defects, including wrinkles, grooves, and even voids, from which stretched portions of the films protrude. These defects locally reduce film thickness and may increase the probability of crack initiation and fragment formation during field use.

Compared to the SEM micrographs of the PE-based mulch film, the biodegradable mulch images (Figure 4b) show a rougher surface, featuring numerous particles in the μm range, which are evenly distributed across the film surface. This dispersed phase corresponds to the semi-crystalline phase of non-plasticized thermoplastic starch granules. Higher-magnification images reveal very fine particles, smaller than 1 μm (Figure 4b, inset). These particles likely correspond to additives included in the mulch film formulations, such as carbon black, inorganic fillers and other additives introduced to improve the compatibility between PBAT and thermoplastic starch. In this respect, the particles are well embedded in the matrix, with no visible signs of delamination or interfacial separation.

The SEM images of C3-PPDE-50-tranparent-0 reveal the characteristic fibrous microstructure of a PP-based nonwoven fleece used for soil protection (Figure 4c). The 200× magnification image shows an interconnected network of PP fibers with diameters of approximately 10–30 μm, forming a porous matrix typical of melt-blown or spunbond nonwoven materials. The inset provides a closer view of fiber junction points, where thermal bonding occurred during manufacturing. These joint regions likely consist of a lower-melting-point polymer, such as PP/PE copolymers with lower crystallinity and melting points, used as a thermal bonding agent. The nonwoven fleece presents significant potential for microfiber release into soil systems, due to fiber diameters falling within the microplastic size range, its high surface area increasing susceptibility to weathering and fragmentation, and the presence of junction points, which may represent weak points where differential thermal expansion and weathering and/or mechanical stress could cause fiber release.

Finally, Figure 4d displays the SEM surface images of SH-BIO2IT-150-white-0, a biodegradable protective sheet for young trees composed of biopolymers and wood scrap fillers. At 200× magnification, the surface appears heterogeneous, with an irregular distribution of both smooth and rough regions. Numerous small, angular particles are embedded within the polymer matrix, consistent with the presence of lignocellulosic biomass or other natural fillers. The visible particulates are likely wood particles, which are well-dispersed but not fully encapsulated by the polymer matrix. This is typical for biocomposite films, where the interface between filler and matrix is often less uniform than in conventional plastics. The underlying matrix shows a relatively continuous phase, suggesting good film formation; however, microvoids and surface roughness are evident. These can be attributed to the inclusion of hydrophilic wood particles and the biodegradable nature of the polymer. The rough, particulate-rich surface and the presence of wood scrap indicate points of weakness, where microbial or enzymatic attack can initiate, thereby enhancing the film’s biodegradability. Compared to fossil-based synthetic shelters, the risk of persistent microplastic release is significantly reduced because both the matrix and the wood filler are biodegradable. Nonetheless, during the early stages of degradation, small fragments or microfibers may still be released, especially from poorly encapsulated wood particles or from brittle regions of the matrix. These fragments are expected to undergo further degradation over time, minimizing long-term accumulation in the soil and supporting the suitability of this material for sustainable agriculture and forestry applications.

#### 3.2.4. Thermogravimetric Analysis

Thermogravimetric analysis (TGA) was used to assess the thermal stability of the selected APs. A summary of the main results is provided here, while a more detailed discussion is available in the Supplementary Material. Table 8 lists the relevant thermal decomposition parameters, including the temperature at which 5% of the initial weight is lost (T_onset_), the peak temperatures (T_peak_) calculated from the derived TG (DTG) curves, the temperature of charring (T_char_), and the weight of the residue at 650 °C (Wt_650_). For all the APs, degradation occurs well above typical processing temperatures, meaning it does not interfere with material use. PE-based APs degraded in a single step between 350 and 480 °C, with I-220-PEEL-1000-black-0 displaying the highest degradation temperature (483.3 °C), likely due to carbon black and other stabilizing additives. PP-based APs showed similar behavior but with slightly lower T_peak_ (425–450 °C), reflecting the PP’s greater susceptibility to thermal degradation (Figure S2). Biodegradable samples exhibited multistep degradation, consistent with the presence of multiple components like starch and polyesters. For instance, the first weight loss of M-BIOFI-15-black-0 at 300 °C (20%) corresponds to starch decomposition, followed by PBAT degradation.

#### 3.2.5. Water Contact Angle (WCA), Swelling Ratio and Water Vapor Permeability of the AP Samples

The characterization of the interaction between APs and both liquid and gaseous water is crucial for ensuring their efficacy, particularly concerning their water swelling, contact angle, and WVP. Water swelling reflects a material’s hydrophilicity, which significantly influences its interaction with moisture and, consequently, its performance in agricultural settings [44]. The contact angle, which quantifies surface wettability, is essential for understanding how water interacts with plastic materials; a lower contact angle typically indicates higher hydrophilicity, which can enhance the material’s moisture uptake and potentially affect its mechanical properties. Furthermore, WVP is a critical parameter, as it determines the ability of agricultural films and covers to regulate water vapor transfer. The interplay among these properties is largely dictated by material formulation. For instance, the addition of plasticizers can modify the hydrophilic characteristics of bioplastics, thereby impacting both contact angle and WVP [45]. These aspects are examined in the following sections, as a comprehensive understanding of these parameters is essential for developing effective APs that meet both functional and sustainability criteria.

##### Water Contact Angle Measurements

A contact angle tester was used to measure the static contact angles of the APs to assess their hydrophobic properties. Wettability is considered high when the contact angle is below 90°, and low when it exceeds 90°. For the examined APs, the contact angle of the M-PEEL-50-transparent-0 film was 79.7°, indicating moderate wetting resistance compared to PE-based mulch films, which exhibit values around 90° [46]. This result can be due to a different polymer matrix formulation, as M-PEEL-50-transparent-0 was likely made up of a polyolefin blend. Furthermore, the presence of additives and fillers also affected WCA. As indicated by TG, the residual char of M-PEEL-50-transparent-0 was 16%. Since fillers are generally more hydrophilic in nature than the PE matrix, higher filler content tends to increase the film’s wettability. M-BIOFI-15-black-0 exhibited a more hydrophobic character, with a WCA of 103.1°. Its residual char was only 5%, which suggests that the hydrophobicity of the PBAT-based blend is higher than that of the PE. Furthermore, the lower wettability also implies that hydrolysis processes could be slowed down, potentially delaying the film’s degradation in soil. Among the other APs, the PP-based samples exhibited a distinctly hydrophobic character, with WCA values of 107.1° and 104.8° for S4-PPEL-1240-green-0 and C3-PPDE-50-white-0, respectively. Interestingly, the biodegradable SH-BIO2IT-150-white-0 shelter displayed the lowest WCA (56.0°), a characteristic that favors faster biodegradation in soil.

##### Swelling Tests

Swelling tests were performed to assess the extent of water absorption in both biodegradable and conventional polyolefin-based samples. The tests were carried out in duplicate. Based on the results (Figure S3), the samples can be grouped into low, medium, and high absorption categories. High absorption was observed for M-BIOFI-15-black-0, which reached values above 100%. Such high water uptake reflects the material’s chemical composition and morphology, clearly distinguishing it from all polyolefin-based plastics. This behavior is typical of biodegradable starch-based blends, which are characterized by a hydrophilic polymer matrix and the presence of hydrophilic fillers or plasticizers [47].

Medium swelling (10–40%) was observed for most products, including O1-PEEL-50-yellow-0 (fly trap), L-PPDE-60-white-0 (low tunnel fleece), C3-PPDE-50-white-0 (protection fleece), R-PEDE-450-green-0 (binding net), S4-PPEL-1240-green-0 (hay bales protection fabric), GT-PPEL-680-green-0 (ground cover fabric), and M-PEEL-50-transparent-0 (mulch film). Although these products are made of hydrophobic polymers (PE and PP), they showed moderate hydrophilicity due to their structure (e.g., O1-PEEL-50-yellow-0, laminated with cellulose) or woven/nonwoven assembly that caused water uptake to increase compared to dense, thin films.

The low swelling group included products that absorbed very little water (<10%), consistent with their hydrophobic nature. As expected, thick and dense PE items like drip tubes and silage or packaging films showed the lowest swelling. Remarkably, the biodegradable shelter SH-BIO2IT-150-white-0 also showed very low absorption (<3%), despite containing hydrophilic lignocellulose filler and having a low WCA. However, SH-BIO2IT-150-white-0 contained a blend of PHAs and PBS polyesters, which are rather hydrophobic and provided the product with remarkable water resistance.

In summary, M-BIOFI-15-black-0 exhibited dramatically higher swelling than polyolefin plastics, making it suitable for applications requiring high water sensitivity and rapid degradation. PE and PP films remain hydrophobic and stable, with minimal swelling, supporting their continued use where resistance to water ingress is needed. However, this also implies the long-term persistence of plastic residues in soil, contributing to microplastic pollution. Woven and nonwoven structures (ground cover, protection fleece) exhibited higher apparent swelling than extruded films. Material thickness also correlates with total swelling (e.g., thicker ground covers swell more than thin films of similar polymer) but remains secondary to the impact of polymer chemistry and structure.

##### Water Vapor Permeability (WVP)

WVP is an important indicator for evaluating the ability of APs to reduce water evaporation [48]. For mulch or tunnel films, high water vapor barrier performance promotes water condensation and improves soil moisture retention, supporting crop growth [49]. Likewise, for container-type products, such as boxes or fertilizer sacks, protecting the contents from external humidity is essential. In this respect, several biodegradable polyesters, such as PBAT, especially when blended with TPS, can be moisture-sensitive, leading to higher WVP. Thus, the water vapor barrier property must be carefully considered when selecting these materials for agricultural applications [50].

The WVP values obtained for the APs (Table 9) reveal clear trends when grouping the samples by polymer type and structural form, reflecting both functional requirements and inherent material properties. PE-based films and fabrics consistently exhibit the lowest WVP values, typically between 0.013 and 0.04 × 10^−13^ g/m·Pa·s for thin films such as mulch films, silage films, and fruit fly traps. This low permeability is characteristic of PE’s dense and semi-crystalline structure, which provides an effective moisture barrier, essential for applications requiring soil moisture retention or protection from ambient humidity (e.g., mulch films and fertilizer sacks) [51]. Woven and nonwoven fabrics used for tree binders, bale protection, and protection fleeces show much higher permeability (from 3.6 to ~26.5 × 10^−13^ g/m·Pa·s) due to their textile-like and porous structure that facilitates vapor transmission. These materials are designed to provide physical protection while allowing vapor exchange, promoting ventilation and reducing condensation risks under covers, to prevent moisture accumulation and related phytopathologies. They also allow moisture to reach the cultivation or soil, reducing irrigation needs. This highlights how plastic permeability can be tailored by modifying thickness and fabric structure to balance moisture retention and breathability.

Biodegradable films and fabrics exhibit intermediate WVP values (approximately 0.6–1.1 × 10^−13^ g/m·Pa·s), which are higher than those of conventional PE films but lower than those of textile-based PP materials. This moderate permeability is typical of biodegradable polyesters, possibly blended with starch or lignocellulosic biomass, which generally have more amorphous and hydrophilic structures that facilitate water vapor diffusion [52,53]. Such permeability supports controlled moisture exchange, potentially improving soil microclimate while also promoting biodegradation. Overall, dense polymer films offer superior moisture–barrier performance, whereas fibrous or biodegradable materials provide enhanced vapor permeability. Material choice therefore reflects a functional compromise between moisture retention and vapor exchange, tailored to specific agricultural functions such as mulching, crop protection, or packaging.

## 4. Conclusions

The findings of this research underline the significant differences between conventional and biodegradable APs in terms of chemical composition, mechanical and physical properties, and overall performance. Conventional plastics, predominantly PE and PP, exhibited high tensile strength, elongation, and favorable radiometric properties, which explain their widespread use in mulching, covering, and geotextile applications. However, these materials also showed no biodegradability, raising concerns about their long-term accumulation in soils and their potential contribution to MNP pollution.

The results demonstrate a clear relationship between the chemical composition, material structure, and functional performance of APs. Conventional polyolefin-based materials (PE and PP), characterized by non-polar hydrocarbon backbones and compact structures, exhibited low water uptake, high mechanical strength, and superior barrier properties. In contrast, biodegradable formulations containing aliphatic polyesters blended with starch and/or lignocellulosic components showed increased polarity and heterogeneous morphologies, resulting in higher water absorption and vapor permeability, lower puncture resistance, and enhanced susceptibility to biodegradation. These findings highlight that the functional behavior of agricultural plastics is governed not only by polymer type but also by the interplay between chemical formulation, additives, and structural organization, providing useful guidelines for the design of materials that balance agronomic performance and environmental sustainability.

The comprehensive characterization carried out in this research highlights that, due to their intrinsic characteristics, oil-based APs leave a long environmental trace. These materials fragment into MNPs that accumulate in soil, alter its properties, and act as carriers for chemicals. Although the biodegradable materials are designed as a more sustainable alternative, their environmental impact remains to be fully assessed. The biodegradable materials are expected to reduce long-term persistence, yet they can still generate fragments during degradation and release chemicals, producing effects that are not yet fully understood. Consequently, we should responsibly try to minimize overall plastic use, prioritize materials with verified field-biodegradation and minimal additive content, and pair their deployment with monitoring programs capable of tracking MNP formation, soil health and interactions with chemicals.

Further research is necessary to ensure that biodegradable plastics meet the functional demands of agriculture while minimizing environmental impacts. The findings of this research contribute to shaping future policies on agricultural plastic use and support the development of more sustainable materials.

## Figures and Tables

**Table 1:** Main properties of the selected APs.

AP Type	Basic Polymer	Manufacturer/Source	Region	AP Characteristics		Code
				Nominal Thickness (μm)	Color	
Mulch film	LLDPE	Eurofilm Matzaris	Korinthia, Greece	50	Transparent	M-PEEL-50-transparent-0
Biodegradable mulch film UNI EN 13432 [10] UNI EN 17033 [11] ASTM 6400 [30]	PBAT, starch	SAMCO	Adare, Ireland	15	Black	M-BIOFI-15-black-0
Geotextile ground cover	PP	Thrace Group	Alimos, Greece	350 (strands are 80 and 40)	Dark green	GT-PPEL-680-green-0
Protection fleece cover	PP	NOOR	Viernheim, Germany	50	White	C3-PPDE-50-white-0
Low tunnel fleece	PP	RL Fundgrube	Gernsheim, Germany	60	White	L-PPDE-60-white-0
Fertilizer sack	LDPE	Thrace Group	Alimos, Greece	150	White	K1-PEEL-250-white-0
Pheromone fly trap	LDPE/paper bilayer	Dakofaka	Iraklio, Greece	100	Yellow	O1-PEEL-50-yellow-0
Drip irrigation pipe	LDPE	Palaplast	Sindos, Greece	1000 (pipe diameter 20 mm)	Black	I-220-PEEL-1000-black-0
Tree binding net	HDPE	RL Fundgrube	Gernsheim, Germany	450 (single strands 50)	Dark Green	R-PEDE-450-green-0
Biodegradable shelter/guide for young trees ISO 17556:2019 [9]	Polyesters, wood scrap	Terralix Sabiomaterials	Forlì, Italy	340	Light beige	SH-BIO2IT-150-white-0
Silage film	LDPE	Polydress SiloPro	Michelstadt, Germany	150	White	S1-PEDE-150-white-0
Hay bales protection fabric	PP	Thrace Group	Alimos, Greece	580 (strands are 50 and 125 mm)	Green	S4-PPEL-1240-green-0

**Table 2:** Main results of Pyrolysis/Thermal Desorption Gas Chromatography–Mass Spectrometry (Py/TD-GC-MS).

Sample Code	Py/TD-GC-MS Results
M-PEEL-50-transparent-0	Degradation products of polyethylene
M-BIOFI-15-black-0	Degradation products of PBAT. Degradation products of starch; cis-13-docosenoamide (slip agent)
GT-PPEL-680-green-0	Degradation products of polypropylene; 2.4 di-tert-butyl-phenol (antioxidants and UV stabilizers)
C3-PPDE-50-white-0	Degradation products of polypropylene; 2.4 di-tert-butyl-phenol (antioxidants and UV stabilizers)
S4-PPEL-1240-green-0	Degradation products of polypropylene; 2.4 di-tert-butyl-phenol (antioxidants and UV stabilizers)
SH-BIO2IT-150-white-0	Degradation products of PHAs. Degradation products of PBS
R-PEDE-450-green-0	Degradation product of polyethylene; 2.4 di-tert-butyl-phenol (antioxidants and UV stabilizers)
L-PPDE-60-white-0	Degradation product of polyethylene; 2.4 di-tert-butyl-phenol (antioxidants and UV stabilizers)
S1-PEDE-150-white-0	Degradation product of polyethylene; 2.4 di-tert-butyl-phenol (antioxidants and UV stabilizers)
I-220-PEEL-1000-black-0	Degradation product of polyethylene; 2.4 di-tert-butyl-phenol (antioxidants and UV stabilizers)
K1-PEEL-250-white-0	Degradation product of polyethylene; 2.4 di-tert-butyl-phenol (antioxidants and UV stabilizers)
O1-PEEL-50-yellow-0	Degradation product of polyethylene; 2.4 di-tert-butyl-phenol (antioxidants and UV stabilizers)

**Table 3:** Metal concentrations of the APs determined using ICP-MS analysis.

	Metals Concentration (mg/kg) *														
AP Sample	Sb	Hg	Pb	Al	Cr	Co	Cu	Zn	As	Cd	Sn	V	Fe	Mn	Ni
M-PEEL-15-black-0	<0.1	<0.1	<0.1	336.5	0.63	<0.1	1.57	46.43	<0.1	<0.1	0.23	<0.1	54.63	2.53	<0.1
M-BIOFI-15-black-0	<0.1	<0.1	<0.1	7.70	<0.1	<0.1	<0.1	2.73	<0.1	<0.1	<0.1	<0.1	18.70	<0.1	<0.1
GT-PPEL-680-green-0	0.79	<0.1	<0.1	14.92	0.18	<0.1	21.21	20.46	<0.1	<0.1	0.17	<0.1	7.29	1.125	0.60
C3-PPDE-50-white-0	<0.1	<0.1	<0.1	2.24	0.29	<0.1	0.10	2.01	<0.1	<0.1	0.16	<0.1	12.31	0.36	<0.1
S4-PPEL-1240-green-0	0.15	<0.1	<0.1	<0.1	<0.1	0.10	11.72	6.14	<0.1	<0.1	0.14	<0.1	3.95	1.36	<0.1
SH-BIO2IT-190-white-0	<0.1	<0.1	0.16	<0.1	0.23	<0.1	0.17	<0.1	<0.1	<0.1	<0.1	<0.1	10.46	7.12	<0.1
R-PEDE-450-green-0	<0.1	<0.1	<0.1	10.77	<0.1	<0.1	2.89	<0.1	<0.1	<0.1	0.15	<0.1	80.03	2.17	<0.1
L-PPDE-60-white-0	<0.1	<0.1	<0.1	4.04	0.48	<0.1	0.96	3.12	<0.1	<0.1	0.13	<0.1	12.21	0.54	0.27
S1-PEDE-150-white-0	<0.1	<0.1	<0.1	4.78	<0.1	<0.1	<0.1	<0.1	<0.1	<0.1	<0.1	<0.1	1.35	0.10	<0.1
I-220-PEEL-1000-black-0	0.20	<0.1	0.41	11.93	<0.1	<0.1	0.95	15.30	<0.1	<0.1	<0.1	<0.1	18.68	0.79	<0.1
K1-PEEL-250-white-0	<0.1	<0.1	<0.1	<0.1	<0.1	<0.1	25.65	<0.1	<0.1	<0.1	0.24	<0.1	4.88	0.22	<0.1
O1-PEEL-50-yellow-0	47.82	<0.1	<0.1	273.9	0.21	<0.1	1.63	42.06	<0.1	<0.1	1.56	<0.1	12.38	0.86	<0.1

* The results are the average of three experimental replicates, with a relative standard deviation of <10%.

**Table 4:** Main absorption peaks found in FTIR-ATR spectra of the analyzed APs.

Sample	Main FTIR Absorption Peaks, cm^−1^												
	3600–3100	2950	2910–2916	2870	2850	2838	1740–1700	1650–1560	1480–1455	1375	1165	1020	740–700
**Conventional and biodegradable mulch films**													
M-PEEL-50-transparent-0	Hydroxyl groups of phenol stabilizers	CH_3_ asymmetrical stretching	CH_2_ asymmetrical stretching	CH_3_ stretching		C–H stretching		CH stretching of phenyl-based stabilizers	CH_3_ symmetrical bending	CH_3_ symmetrical bending	C–H, CH_3_ wagging, rocking		CH_2_ rocking
M-BIOFI-15-black-0	Terminal hydroxyl groups				CH_2_ symmetrical stretching in PBAT		C=O stretching due to ester bonds in PBAT			1270 cm^−1^, C–O stretching of aromatic esters			
**Geotextile ground cover**													
GT-PPEL-680-green-0	Hydroxyl groups of phenol stabilizers	CH_3_ asymmetrical stretching	CH_2_ asymmetrical stretching	CH_3_ stretching		C–H stretching		CH stretching of phenyl-based stabilizers	CH_3_ symmetrical bending	CH_3_ symmetrical bending	C–H, CH_3_ wagging, rocking		
**Protection fleece cover**													
C3-PPDE-50-white-0	Hydroxyl groups of phenol stabilizers	CH_3_ asymmetrical stretching	CH_2_ asymmetrical stretching	CH_3_ stretching		C–H stretching		CH stretching of phenyl-based stabilizers	CH_3_ symmetrical bending	CH_3_ symmetrical bending	C–H, CH_3_ wagging, rocking		
**Hay bale protection fabric**													
S4-PPEL-1240-green-0	Hydroxyl groups of phenol stabilizers and plasticizers	CH_3_ asymmetrical stretching	CH_2_ asymmetrical stretching	CH_3_ stretching		C–H stretching	C=O stretching due to plasticizers		CH_3_ symmetrical bending	CH_3_ symmetrical bending	C–H, CH_3_ wagging, rocking		
**Tree binding net**													
R-PEDE-450-green-0	Hydroxyl groups of phenol stabilizers		CH_2_ asymmetrical stretching		CH_2_ symmetrical stretching in PE				CH_2_ bending				CH_2_ rocking
**Biodegradable shelter/guide for young trees**													
SH-BIO2IT-150-white-0	Hydroxyl groups of terminal groups	C–H, CH_2_ symmetrical stretching					C=O stretching due to ester bonds in PHAs, PES/PBS			1270 cm^−1^, C–O stretching of aromatic esters		C–O stretching due to starch	727 cm^−1^, C–H deformation
**Low tunnel fleece**													
L-PPDE-60-white-0	Hydroxyl groups of phenol stabilizers	CH_3_ asymmetrical stretching	CH_2_ asymmetrical stretching	CH_3_ stretching		C–H stretching			CH_3_ symmetrical bending	CH_3_ symmetrical bending	C–H, CH_3_ wagging, rocking		
**Silage film**													
S1-PEDE-150-white-0	Hydroxyl groups of phenol stabilizers		CH_2_ asymmetrical stretching		CH_2_ symmetrical stretching in PE		C=O stretching due to plasticizers		CH_2_ bending				CH_2_ rocking
**Drip irrigation pipe**													
I-220-PEEL-1000-black-0	Hydroxyl groups of phenol stabilizers		CH_2_ asymmetrical stretching		CH_2_ symmetrical stretching in PE				CH_2_ bending				CH_2_ rocking
**Fertilizer sack**													
K1-PEEL-250-white-0			CH_2_ asymmetrical stretching		CH_2_ symmetrical stretching in PE				CH_2_ bending				CH_2_ rocking
**Pheromone fly trap**													
O1-PEEL-50-yellow-0			CH_2_ asymmetrical stretching		CH_2_ symmetrical stretching in PE			CH stretching of phenyl-based stabilizer	CH_2_ bending				CH_2_ rocking

**Table 5:** Tensile properties of the analyzed AP samples.

Sample	Thickness (μm)	Mechanical Properties *			
	MD	Tensile Strength (MPa)		Elongation at Break (%)	
	TD	MD	TD	MD	TD
**Conventional and biodegradable mulch films ^§^**					
M-PEEL-50-transparent-0	44.6 ± 1.1	23.4 ± 3.0 ^b^	21.3 ± 1.0 ^b^	390.1 ± 26.4 ^c^	502.5 ± 13.9 ^b^
	44.4 ± 0.6				
M-BIOFI-15-black-0	13.2 ± 1.8	21.8 ± 2.8 ^b,c^	16.8 ± 2.6 ^c^	293.3 ± 17.2 ^d^	388.8 ± 32.5 ^c^
	11.6 ± 0.9				
Sample	Thickness (μm) MD/TD (only MD direction for tubes)	Mechanical Properties			
		Tensile Strength (MPa)		Elongation at Break (%)	
		MD	TD	MD	TD
**Drip irrigation pipe ^§^**					
I-220-PEEL-1000-black-0	1110 ± 40	26.8 ± 3.0 ^b^	N/A	1049.3 ± 76.9 ^a^	N/A
**Fertilizer sack, Silage film, Pheromone fly trap ^§^**					
K1-PEEL-250-white-0	147.7 ± 5.0	34.6 ± 3.3 ^a^	31.0 ± 1.0 ^a^	652.8 ± 45.4 ^b^	679.8 ± 5.1 ^a^
	143.7 ± 2.6				
S1-PEDE-150-white-0	142.9 ± 2.2	24.2 ± 2.1 ^b^	25.6 ± 2.5 ^b^	574.3 ± 46.1 ^b^	671.6 ± 50.5 ^a^
	141.5 ± 0.9				
O1-PEEL-50-yellow-0	93.4 ± 1.4	33.0 ± 1.5 ^a^	30.5 ± 2.3 ^a,b^	93.7 ± 10.1 ^f^	65.3 ± 18.0 ^e,f^
	96.0 ± 1.5				
**Protection fleece cover, geotextile ground cover, low tunnel fleece, conventional mulching fabric, tree binding net ^#^**					
Sample	Single Nominal Thickness (μm)	Mechanical Properties			
		Tensile Strength (N/mm)		Elongation at Break (%)	
		Direction 1	Direction 2	Direction 1	Direction 2
C3-PPDE-50-white-0	50	0.40 ± 0.03 ^e^	0.60 ± 0.04 ^d^	193.20 ± 50.30 ^e^	98.60 ± 21.00 ^e^
GT-PPEL-680-green-0	350	18.20 ± 1.20 ^c^	20.80 ± 1.20 ^b,c^	49.00 ± 2.70 ^h^	37.90 ± 10.90 ^f^
L-PPDE-60-white-0	60	0.20 ± 0.03 ^f^	0.30 ± 0.05 ^e^	183.90 ± 10.00 ^e^	171.90 ± 15.70 ^d^
R-PEDE-450-green-0	450	11.80 ± 0.50 ^d^	N/A	39.30 ± 12.10 ^h^	N/A
**Hay bale protection fabric ^^^**					
Sample	Single Nominal Thickness (μm)	Mechanical Properties			
		Tensile Strength (N/mm)		Elongation at Break (%)	
		MD	TD	MD	TD
S4-PPEL-1240-green-0	580	33.10 ± 3.20 ^a^	34.40 ± 2.20 ^a^	59.20 ± 5.90 ^g^	62.20 ± 18.50 ^e,f^
**Biodegradable shelter/guide for young trees ^§,^^**					
Sample	Thickness (μm)	Mechanical Properties			
		Tensile Strength (MPa)		Elongation at Break (%)	
		MD	TD	MD	TD
SH-BIO2IT-150-white-0 ^#^	337 ± 13	21.30 ± 2.50 ^b,c^	18.30 ± 2.20 ^b^	6.50 ± 0.60 ^i^	5.20 ± 2.00 ^g^

(*) MD: machine direction; TD: transverse direction. (§) Tested with typical grips. (#) Tested with grips for agricultural nets (not discrete directions; named directions 1 and 2). (^) Tested with grips for agricultural nets (discrete directions). Values sharing the same superscript letter are not significantly different.

**Table 6:** Puncture properties of the analyzed AP samples.

Sample	Nominal Thickness (mm)	Maximum Load (N)	Displacement (mm)
M-PEEL-50-transparent-0	50	34.3 ± 1.0 ^a^	20.4 ± 0.6 ^d^
M-BIOFI-15-black-0	15	12.1 ± 2.3 ^c^	14.7 ± 2.5 ^e^
SH-BIO2IT-150-white-0	340	2.4 ± 1.2 ^d^	12.9 ± 1.5 ^e^
S4-PPEL-1240-green-0	58	4.4 ± 1.4 ^d^	35.6 ± 6.2 ^c^
K1-PEEL-250-white-0	150	10.2 ± 4.3 ^c^	59.2 ± 5.2 ^a^
O1-PEEL-50-yellow-0	100	25.4 ± 3.5 ^b^	44.4 ± 2.7 ^b^

Values sharing the same superscript letter are not significantly different.

**Table 7:** Radiometric properties of conventional and biodegradable AP samples. PAR: photosynthetic active radiation; LWIR: long-wave infrared.

Sample	Solar Total Transmissivity %	Solar Diffuse Transmissivity %	PAR Total Transmissivity %	PAR Diffuse Transmissivity %	LWIR Transmissivity %	LWIR Emissivity %
M-PEEL-50-transparent-0	89.6 ± 1.6 ^a^	89.6 ± 1.6 ^a^	89.5 ± 1.6 ^a^	89.5 ± 1.6 ^a^	36.0 ± 0.5 ^d^	57.6 ± 1.2 ^g^
M-BIOFI-15-black-0	4.9 ± 0.4 ^f^	3.1 ± 0.3 ^h^	1.7 ± 0.2 ^f^	1.6 ± 0.2 ^g^	16.2 ± 0.1 ^g^	80.1 ± 0.4 ^d^
K1-PEEL-250-white-0	52.5 ± 0.0 ^c^	44.5 ± 0.0 ^e^	45.3 ± 0.0 ^c^	45.0 ± 0.0 ^c^	52.7 ± 0.9 ^b^	41.2 ± 1.3 ^i^
S1-PEDE-150-white-0	37.7 ± 0.0 ^e^	34.0 ± 0.0 ^f^	27.6 ± 0.0 ^e^	27.5 ± 0.0 ^e^	51.6 ± 0.1 ^c^	43.7 ± 0.0 ^h^
O1-PEEL-50-yellow-0	47.7 ± 0.4 ^d^	46.8 ± 0.0 ^d^	39.0 ± 0.3 ^d^	38.4 ± 0.0 ^d^	0.7 ± 0.1 ^j^	97.0 ± 0.0 ^b^
C3-PPDE-50-white-0	87.1 ± 2.5 ^a^	53.5 ± 2.1 ^c^	86.8 ± 2.6 ^a^	53.0 ± 2.1 ^b^	23.5 ± 1.0 ^f^	75.0 ± 1.0 ^e^
GT-PPEL-680-green-0	0.9 ± 0.1 ^g^	0.9 ± 0.0 ^i^	0.2 ± 0.0 ^g^	0.2 ± 0.0 ^h^	9.2 ± 0.5 ^h^	89.0 ± 0.4 ^c^
S4-PPEL-1240-green-0	0.4 ± 0.1 ^h^	0.3 ± 0.0 ^j^	0.0 ± 0.0 ^h^	0.0 ± 0.0 ^i^	1.5 ± 0.0 ^i^	97.1 ± 0.1 ^b^
L-PPDE-60-white-0	89.6 ± 4.9 ^a^	50.0 ± 4.5 ^c,d^	89.7 ± 4.6 ^a^	49.9 ± 4.5 ^b^	32.9 ± 0.3 ^e^	65.7 ± 0.3 ^f^
R-PEDE-450-green-0	52.1 ± 0.5 ^c^	11.8 ± 0.7 ^g^	45.1 ± 0.4 ^c^	9.1 ± 0.4 ^f^	54.3 ± 0.8 ^a^	44.4 ± 0.7 ^h^
SH-BIO2IT-150-white-0	69.7 ± 2.6 ^b^	59.8 ± 1.0 ^b^	61.2 ± 3.6 ^b^	56.4 ± 2.8 ^b^	0.1 ± 0.0 ^k^	97.9 ± 0.0 ^a^

Values sharing the same superscript letter are not significantly different.

**Table 8:** Main thermal parameters of the analyzed AP samples as measured by thermogravimetry.

Sample	T_onset, W 5%_ (°C)	T_peak1_ (°C)	T_peak2_ (°C)	T_peak3_ (°C)	T_char_ (°C)	Wt_650_ (%)
M-PEEL-50-transparent-0	355.0 ± 2.1 ^f^	-	352.0 ± 1.8 ^c^	477.0 ± 2.5 ^b^	531.0 ± 3.2 ^c^	16.0 ± 1.5 ^a^
M-BIOFI-15-black-0	289.0 ± 2.3 ^h^	300.0 ± 1.9 ^a^	396.0 ± 2.1 ^a^	496.0 ± 2.8 ^a^	537.0 ± 3.5 ^b^	5.0 ± 0.8 ^b^
R-PEDE-450-green-0	397.0 ± 1.8 ^c^	-	-	467.4 ± 2.2 ^c^	472.4 ± 2.9 ^d^	0.8 ± 0.1 ^c^
SH-BIO2IT-150-white-0	251.7 ± 2.5 ^i^	264.9 ± 2.0 ^b^	389.9 ± 2.3 ^b^	-	646.9 ± 4.1 ^a^	8.0 ± 1.0 ^d^
S4-PPEL-1240-green-0	387.1 ± 2.0 ^d^	-	-	458.1 ± 2.1 ^f^	648.0 ± 3.8 ^a^	3.0 ± 0.6 ^d^
S1-PEDE-150-white-0	404.2 ± 1.9 ^b^	-	-	459.8 ± 2.3 ^e^	645.0 ± 3.6 ^a^	14.0 ± 1.2 ^e^
O1-PEEL-50-yellow-0	247.2 ± 2.8 ^j^	-	358.9 ± 2.4 ^c^	470.8 ± 2.6 ^c^	648.0 ± 4.0 ^a^	7.0 ± 0.9 ^f^
K1-PEEL-250-white-0	381.7 ± 2.0 ^e^	-	-	476.7 ± 2.4 ^b^	645.7 ± 3.7 ^a^	0.3 ± 0.1 ^g^
I-220-PEEL-1000-black-0	443.3 ± 1.7 ^a^	-	-	483.3 ± 2.1 ^b^	650.0 ± 3.5 ^a^	2.0 ± 0.5 ^h^
GT-PPEL-680-green-0	337.3 ± 2.4 ^g^	-	-	425.8 ± 2.7 ^h^	647.9 ± 3.9 ^a^	7.0 ± 0.8 ^i^
C3-PPDE-50-white-0	355.1 ± 2.2 ^f^	-	-	450.3 ± 2.3 ^g^	480.0 ± 3.0 ^d^	0.9 ± 0.1 ^j^
L-PPDE-60-white-0	354.2 ± 2.3 ^f^	-	-	439.9 ± 2.5 ^i^	642.0 ± 4.2 ^a^	0.3 ± 0.1 ^j^

Values sharing the same superscript letter are not significantly different.

**Table 9:** Water Contact Angle, swelling ratio after 24 h, and water vapor permeability of the AP samples.

Sample	WCA (°)	Swelling Ratio (%)	WVP (g/m·Pa·s) (×10^−13^)
M-PEEL-50-transparent-0	79.70 ± 2.90 ^c^	9.07 ± 7.03 ^d^	0.02 ± 0.01 ^g,h^
M-BIOFI-15-black-0	103.10 ± 3.40 ^a^	101.60 ± 22.30 ^a^	0.63 ± 0.03 ^f^
SH-BIO2IT-150-white-0	56.00 ± 3.10 ^e^	2.08 ± 0.62 ^e^	1.11 ± 0.16 ^e^
R-PEDE-450-green-0	-	20.85 ± 3.68 ^c^	26.50 ± 0.30 ^a^
S4-PPEL-1240-green-0	107.10 ± 3.60 ^a^	23.22 ± 4.70 ^c^	17.30 ± 0.60 ^b^
S1-PEDE-150-white-0	94.30 ± 3.00 ^b^	1.57 ± 0.38 ^e^	0.04 ± 0.01 ^g^
L-PPDE-60-white-0	87.20 ± 4.60 ^b,c^	40.34 ± 3.40 ^b^	4.89 ± 1.01 ^d^
GT-PPEL-680-green-0	84.00 ± 2.00 ^b,c^	8.46 ± 3.77 ^d^	11.00 ± 0.70 ^c^
O1-PEEL-50-yellow-0	73.40 ± 7.00 ^c,d^	40.05 ± 1.69 ^b^	0.01 ± 0.002 ^h^
K1-PEEL-250-white-0	67.00 ± 3.10 ^d^	1.70 ± 0.59 ^e^	0.02 ± 0.002 ^g^
I-220-PEEL-1000-black-0	-	7.56 ± 6.68 ^d^	-
C3-PPDE-50-white-0	104.80 ± 5.80 ^a^	22.62 ± 2.84 ^c^	3.59 ± 0.07 ^d^

Values sharing the same superscript letter are not significantly different.

## Data Availability

The original contributions presented in this study are included in the article. Further inquiries can be directed to the corresponding authors.

## Supplementary Materials

The following supporting information can be downloaded at: https://www.mdpi.com/article/10.3390/polym18131656/s1, Figure S1: FTIR spectra of the 12 selected AP materials (AP samples name are given within each spectrum); Figure S2: TG curves and DTG of M-PEEL-50-transparent-0, M-BIOFI-15-black-0, SH-BIO2IT-150-white-0, and GT-PPEL-680-green-0; Figure S3: Swelling in water of the tested AP samples; Table S1: Structural assignment of the macromolecular adducts in the mass spectra reported in Figure 3; Table S2: Structural assignment of the macromolecular adducts in the mass spectra of SH-BIO2IT-150-white-0, reported in Figure 2B; Table S3: Main thermal parameters of the analyzed AP samples as measured by thermogravimetry; Refs. [54,55] are cited in Supplementary Materials File.
