# Supplementary material for: Holistic Approach for the Comparative Assessment of Chemical Structure and Functional Properties of Major Categories of Agricultural Plastics
Source: Polymers (Basel). 2026 Jul 3;18(13):1656. doi: 10.3390/polym18131656 (PMC13363739; doi:10.3390/polym18131656)
Supplement: Supplementary file 1 [file polymers-18-01656-s001.zip › polymers-4290860-supplementary.pdf]

Supporting Information

Comparative Assessment of Chemical Structure and Functional Properties of Major Categories of Agricultural Plastics

Table S1. Structural assignment of the macromolecular adducts in the mass spectra reported in Figure 3.

**symbol**	**Copolymer Structure**	**Na⁺** *m/z*
4A/1T	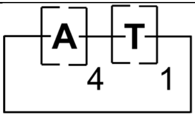	1043.710
3A/2T	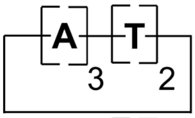	1063.607
2A/3T	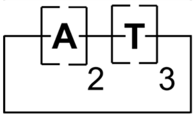	1083.536
1A/4T	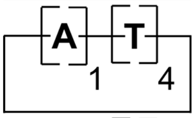	1103.421
4A/2T	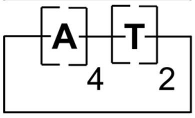	1263.741
3A/3T	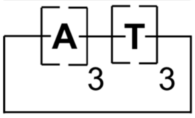	1283.709
2A/4T	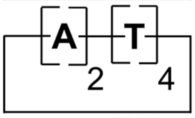	1303.652
1A/5T	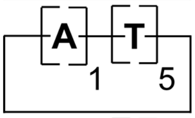	1323.642
5A/2T	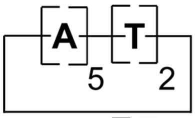	1463.864
4A/3T	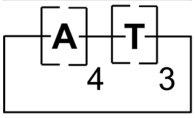	1483.828
3A/4T	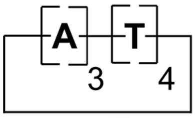	1503.752
2A/5T	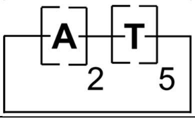	1523.728

A= 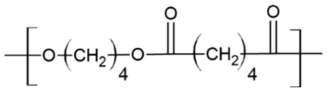

T= 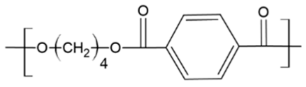

Table S2. Structural assignment of the macromolecular adducts in the mass spectra of SH-BIO2IT-150-white-0, reported in Figure 2B.

**symbol**	**Copolymer Structure**	**Na^+** *m/z*	**K^+** *m/z*
9PBS	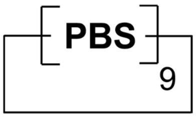	1571.938	1587.231
8PBS	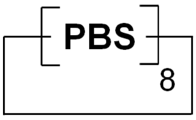	1399.848	1415.840
7PBS	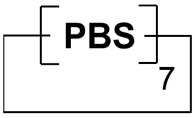	1227.748	1243.723
6PBS	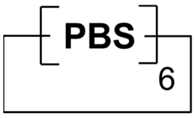	1055.647	1071.646
5PBS	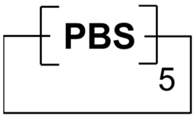	883.547	899.528

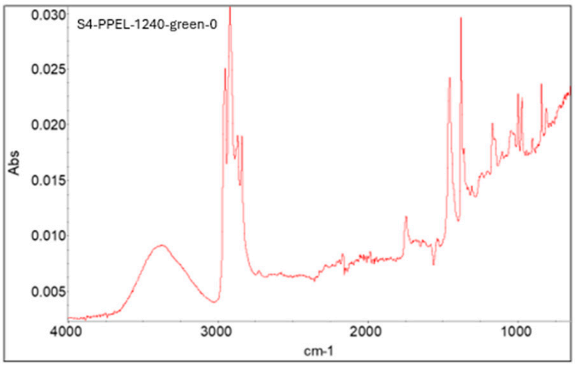
S4-PPEL-1240-green-0
Abs
0.030
0.025
0.020
0.015
0.010
0.005
4000
3000
2000
1000
cm-1

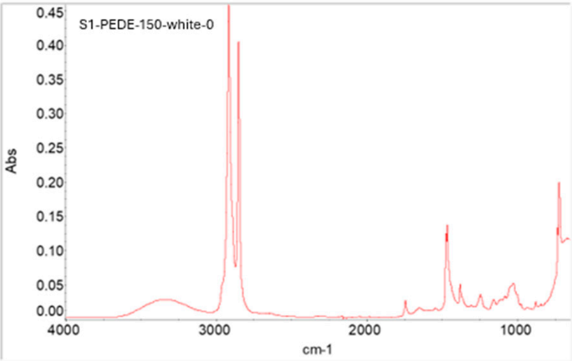
S1-PEDE-150-white-0
Abs
0.45
0.40
0.35
0.30
0.25
0.20
0.15
0.10
0.05
0.00
4000
3000
2000
1000
cm-1

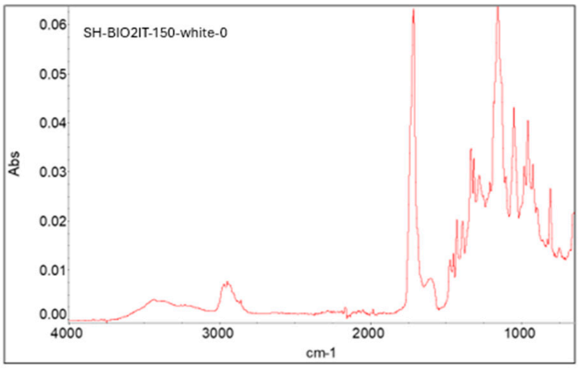
SH-BIO2IT-150-white-0
Abs
0.06
0.05
0.04
0.03
0.02
0.01
0.00
4000
3000
2000
1000
cm-1

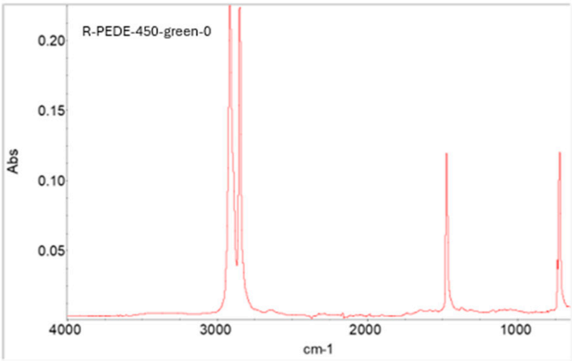
R-PEDE-450-green-0
Abs
0.20
0.15
0.10
0.05
4000
3000
2000
1000
cm-1

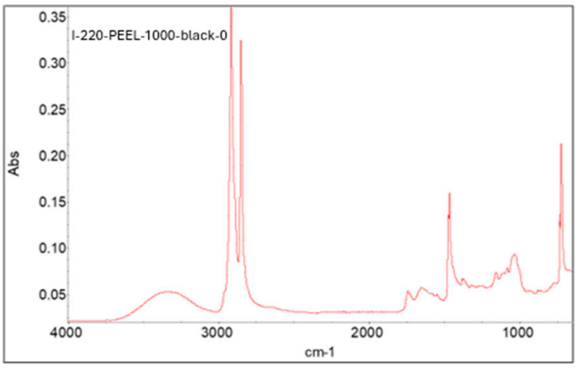
I-220-PEEL-1000-black-0
Abs
0.35
0.30
0.25
0.20
0.15
0.10
0.05
4000
3000
2000
1000
cm-1

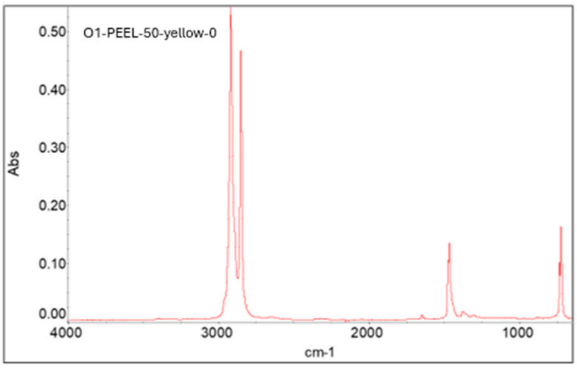
O1-PEEL-50-yellow-0
Abs
0.50
0.40
0.30
0.20
0.10
0.00
4000
3000
2000
1000
cm-1

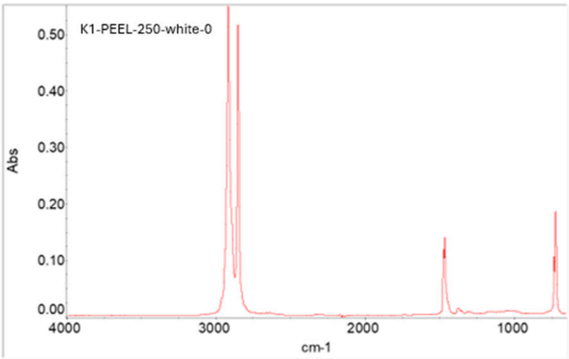
K1-PEEL-250-white-0
Abs
0.50
0.40
0.30
0.20
0.10
0.00
4000
3000
2000
1000
cm-1

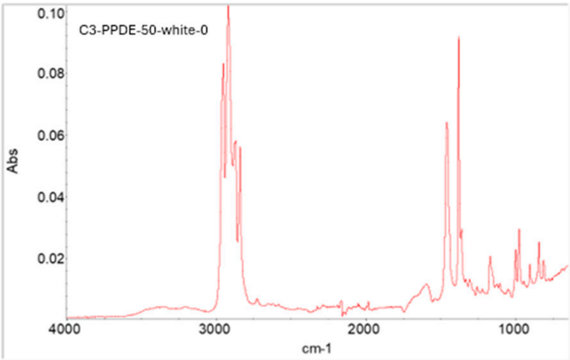
C3-PPDE-50-white-0
Abs
0.10
0.08
0.06
0.04
0.02
4000
3000
2000
1000
cm-1

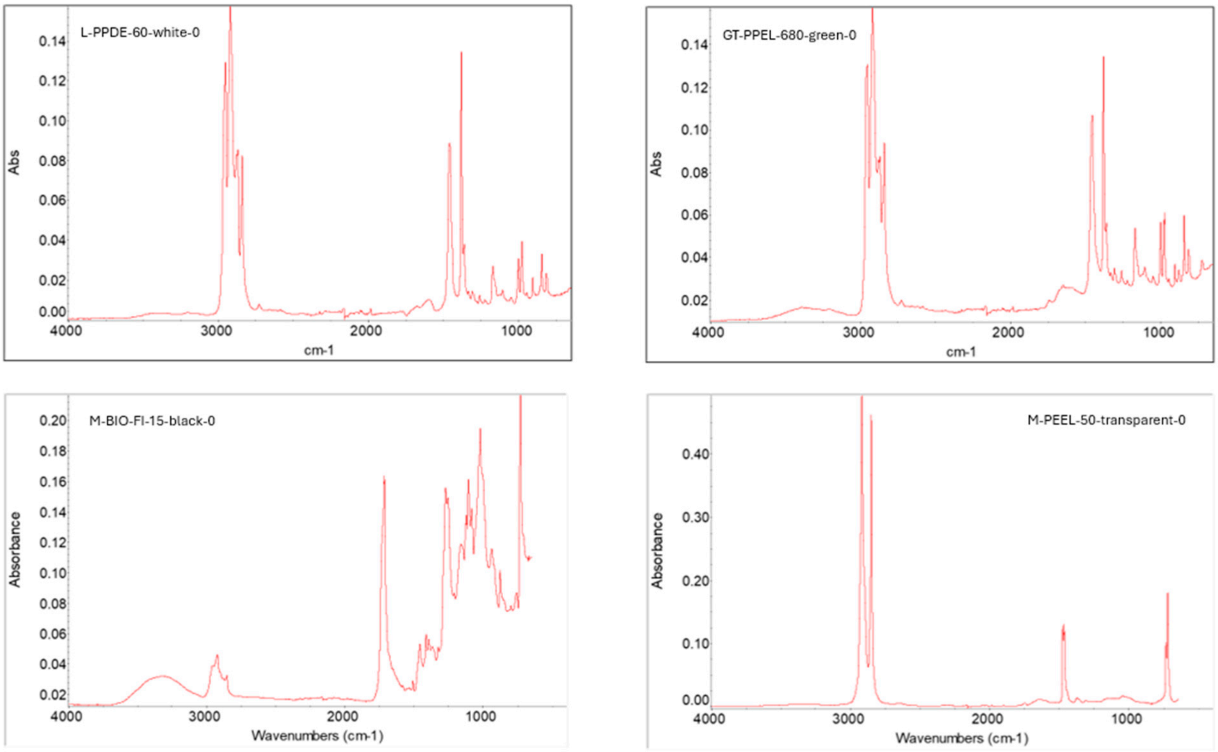

Figure S1. FTIR spectra of the 12 selected AP materials (AP sample names are given within each spectrum).

Thermogravimetric Analysis

Thermogravimetric curves reveal the weight loss as a function of temperature resulting from liquid or solid gaseous phase transitions, providing information on the composition and thermal stability of pure polymer substances or blends. Herein, thermogravimetry was used to gain further insight into the stability of the selected agricultural plastics (APs). **Figure S2** shows the thermograms and the DTG derivative curves of some selected APs based on conventional and biodegradable polymers. **Table S3** summarizes the relevant thermal decomposition parameters calculated, including the temperature at which 5% of the initial weight is lost (T_{onset}), the peak temperatures (T_{peak}) calculated from the DTG curves, the temperature of charring (T_{char}), and the weight of the residue at 650 °C (Wt_{650}). From the table, it is noted that most of polyethylene-based APs degrade in a single weight loss step, at temperatures between 450°C and 480°C, which correspond to the reported polyethylene degradation peak temperatures. More specifically, the thicker the sample, the higher the T_{peak3} temperature, as displayed by I-220-PEEL-1000-black-0 (483.3 °C). Interestingly, R-PEDE-450-green-0, which is made of HDPE, shows a quite high T_{peak3} temperature of 467.4 °C, even though the thickness of the single strands is 50 µm. M-PEEL-50-transparent-0 mulch film shows a degradation behavior made up of two consecutive

weight-loss steps: the first one at 352 °C, responsible for a weight loss of about 7.5%, and the second peaked at 477 °C (**Figure S1**). This latter corresponds to polyethylene degradation temperature, while the presence of the low temperature degradation phenomenon suggests the presence of a highly branched polymer matrix. This makes it a lighter, ductile, and flexible material, making it ideal for producing films, used for agricultural mulching. O1-PEEL-50-yellow-0 also shows two peaks: the second at 470.8 °C is due to LDPE degradation, while the first one (358.9 °C) is related to the presence of cellulose in the LDPE/paper bilayered structure.

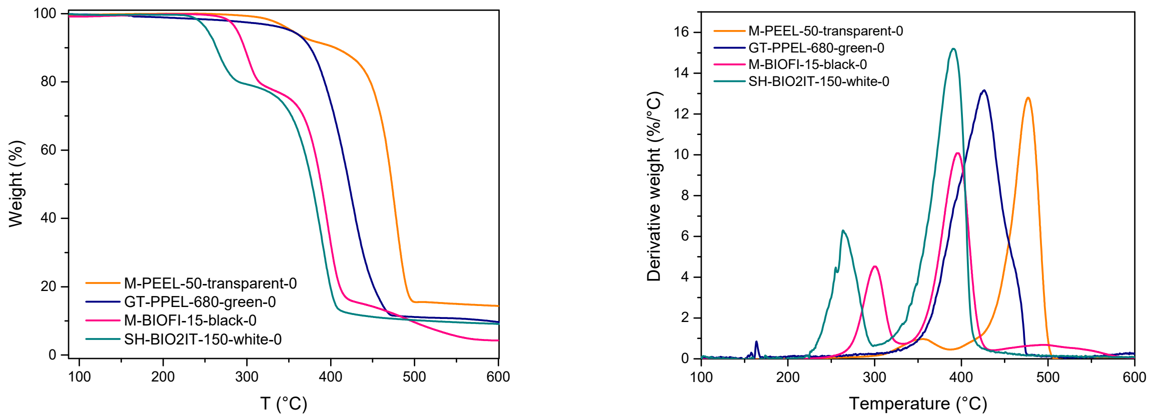

Figure S2. TG curves and DTG of M-PEEL-50-transparent-0, M-BIOFI-15-black-0, SH-BIO2IT-150-white-0, and GT-PPEL-680-green-0.

Table S3. Main thermal parameters of the analyzed AP samples as measured by thermogravimetry.

Sample	$T_{onset, W 5\%}$ (°C)	T_{peak1} (°C)	T_{peak2} (°C)	T_{peak3} (°C)	T_{char} (°C)	Wt_{650} (%)
M-PEEL-50-transparent-0	355.0	-	352.0	477.0	531.0	16.0
M-BIOFI-15-black-0	289.0	300.0	396.0	496.0	537.0	5.0
R-PEDE-450-green-0	397.0	-	-	467.4	472.4	0.8
SH-BIO2IT-150-white-0	251.7	264.9	389.9	-	646.9	8.0
S4-PPEL-1240-green-0	387.1	-	-	458.1	648.0	3.0
S1-PEDE-150-white-0	404.2	-	-	459.8	645.0	14.0
O1-PEEL-50-yellow-0	247.2	-	358.9	470.8	648.0	7.0
K1-PEEL-250-white-0	381.7	-	-	476.7	645.7	0
I-220-PEEL-1000-black-0	443.3	-	-	483.3	650.0	2.0
GT-PPEL-680-green-0	337.3	-	-	425.8	647.9	7.0
C3-PPDE-50-white-0	355.1	-	-	450.3	480.0	0.0
L-PPDE-60-white-0	354.2	-	-	439.9	642.0	0.3

Polypropylene-based APs exhibit a similar trend, but with a slightly lower T_{peak} ranging from 425°C to 450 °C, as expected for polypropylene [1], which is more prone to thermal degradation of PE due to the presence of tertiary carbon atoms.

Concerning the biodegradable samples, both of them show a multistep degradation pattern, suggesting the presence of different components in a polyester-based formulation. For M-BIOFI-15-black-0, the first degradation step occurs at 300 °C, with a weight loss of about 20%. This step is related to the thermal decomposition of starch, which is followed by the degradation of the PBAT matrix [2]. On the other hand, SH-BIO2IT-150-white-0 was less stable (T_{peak1} = 265 °C), indicative of a lignocellulose filler with high hemicellulose and extractives content, such as corn husks, sugarcane bagasse, or wheat straw [3]. The second step is consistent with the degradation step of a polyester matrix, such as a blend of PBS/PHA. Nevertheless, it is worth mentioning that the degradation of the samples occurs well above the processing temperature range, thus not affecting their utilization.

The charring step mainly reflects the stability and the amount of additives included in the formulation. Therefore, the evaluation of the charring temperature and residual weight of the mulch film samples provides a convenient method for determining the amount of carbon black and the content of other inorganic additives, as they are not degraded under nitrogen at 600 °C. From **Table S3**, it is seen that M-PEEL-50-transparent-0 contained a higher amount of filler with respect to M-BIOFI-15-black-0. In particular, considering the results of the ICP-MS characterization, calcium carbonate or aluminum oxide (alumina) are likely present alongside carbon black, as even small amounts of the latter (usually less than 3%) are capable of producing a consistent black color. The highest char value (about 16%) was found for M-PEEL-50-transparent-0. In this film, no carbon black was used; therefore, charring is likely due to other fillers, including calcium carbonate, silica, or alumina. A higher content of fillers perturbs the polymer chain interactions, leading to the formation of defects in the polymer film, and therefore is indicative of a higher tendency to fragmentation and release of micro- and nano-plastics in soil.

Swelling tests

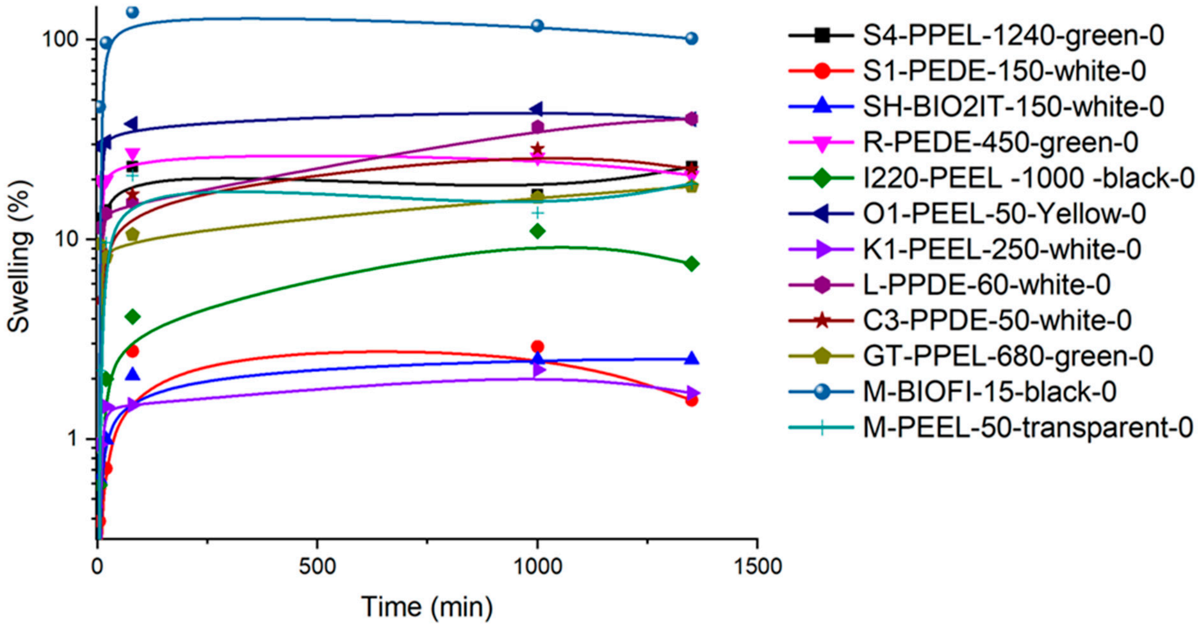

Figure S3. Swelling in water of the tested AP samples.

References

1. Esmizadeh, E., Tzoganakis, C., & Mekonnen, T. H. (2020). Degradation behavior of polypropylene during reprocessing and its biocomposites: Thermal and oxidative degradation kinetics. *Polymers*, *12*(8), 1627. doi: 10.3390/polym12081627
2. Convertino, F., Carroccio, S.C., Cocca, M.C., Dattilo, S., Dell'Acqua, A.C., Gargiulo, L., Nizzetto, L., Riccobene, P.M., Schettini, E., Vox, G., Zannini, D., Cerruti, P., 2024. The fate of post-use biodegradable PBAT-based mulch films buried in agricultural soil. Sci. Total Environ. 948, 174697. https://doi.org/10.1016/J.SCITOTENV.2024.174697
3. Pešenjanski, I., Miljković, B., & Vićević, M. (2016). Pyrolysis kinetic modelling of wheat straw from the pannonian region. Journal of Combustion, 2016(1), 9534063. https://doi.org/10.1155/2016/9534063
